# Inkjet Printing of Functional Materials for Optical and Photonic Applications

**DOI:** 10.3390/ma9110910

**Published:** 2016-11-10

**Authors:** Jorge Alamán, Raquel Alicante, Jose Ignacio Peña, Carlos Sánchez-Somolinos

**Affiliations:** 1 Departamento de Física de la Materia Condensada, Instituto de Ciencia de Materiales de Aragón (ICMA), CSIC-Universidad de Zaragoza, C./Pedro Cerbuna 12, Zaragoza 50009, Spain; jorge.alaman@bshg.com (J.A.); raquela@unizar.es (R.A.); 2 BSH, Polígono Industrial de PLA-ZA, Ronda del Canal Imperial de Aragón, 18-20, Zaragoza 50197, Spain; 3 Departamento de Ciencia y Tecnología de Materiales y Fluidos, Instituto de Ciencia de Materiales de Aragón (ICMA), CSIC-Universidad de Zaragoza, C./María de Luna 3, Zaragoza 50018, Spain; jipena@unizar.es

**Keywords:** inkjet printing, functional materials, optical applications, photonic devices, microlenses, OLEDs, solar cells, liquid crystals, colloidal crystals, sensors

## Abstract

Inkjet printing, traditionally used in graphics, has been widely investigated as a valuable tool in the preparation of functional surfaces and devices. This review focuses on the use of inkjet printing technology for the manufacturing of different optical elements and photonic devices. The presented overview mainly surveys work done in the fabrication of micro-optical components such as microlenses, waveguides and integrated lasers; the manufacturing of large area light emitting diodes displays, liquid crystal displays and solar cells; as well as the preparation of liquid crystal and colloidal crystal based photonic devices working as lasers or optical sensors. Special emphasis is placed on reviewing the materials employed as well as in the relevance of inkjet in the manufacturing of the different devices showing in each of the revised technologies, main achievements, applications and challenges.

## 1. Introduction

Printing methods which allow inks to be precisely deposited have traditionally been used to reproduce written text and graphic works. Printing techniques have strongly fuelled information and knowledge conservation and spreading, ensuring the survival of ideas decisively contributing to progress of civilisation [1]. From cave painting to modern digital printing technologies, the development of ink deposition techniques has significantly evolved through centuries trying to adapt to existing needs and exploiting technological developments of each period. Among these technologies, inkjet printing has gained interest over the last decades due to its ability to digitally control the ejection of ink droplets of defined volume and precisely position them onto a substrate. Compared to analogical methodologies such as flexography, in which the use and preparation of complicated printing plates are needed, digital inkjet printing allows for the quick preparation of different designs with reduced cost becoming a very flexible tool for customisation.

**Figure 1:**
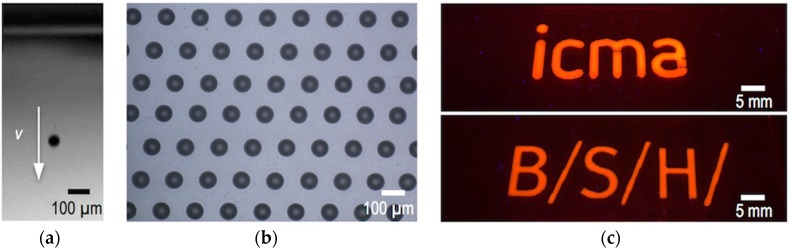
Inkjet printing process: (**a**) Picoliter volume ink droplets are generated and fly towards the substrate at speed v; (**b**) Droplets are precisely deposited onto a surface; (**c**) Inclusion of functional materials (e.g., luminescent) leads to functional prints or structures.

Although the traditional and most wide use of inkjet printing has been centred in conventional applications such as graphics, text printing or marking, the ability to accurately position picoliter volumes of a large range of materials under digital control has been more recently exploited to pattern and manufacture novel functional surfaces and devices (Figure 1). Patterning at small length scales of materials with semiconducting, conducting, luminescent or magnetic functionalities has demonstrated to be a key element to implement emerging technologies and applications such as electronic devices or displays, sensors, bio-arrays, radio frequency identifiers (RFIDs) or solar cells [2,3,4].

Being an additive method, all the deposited material, except volatile carriers or by-products, is used in the final patterned surface or device, not needing any additional etching step and therefore using materials very efficiently. As a result, inkjet printing is an environmentally friendly technique. Besides, inkjet printing is a non-contact method so there is a large flexibility in the use of substrates of different materials, sizes, even non-flat or flexible ones. Despite inkjet printing is a serial process, it is a scalable technology able to deliver high throughput. For example, multiple printheads, each having a large number of nozzles ejecting at very high frequencies can be used, allowing multi-ink wide print-width with a single pass leading to a high productivity. Differently from other conventional printing processes, inkjet printing, as a digital technique, enables in-line correction of defects or distortions, by automatically adjusting the printing configuration of nozzles when a defect is detected, what is critical to obtain productions at high yield in certain defect-sensitive applications. In addition, inkjet printing is compatible with roll to roll (R2R) processing and can be effectively combined with other existing techniques such as gravure, screen-printing, offset lithography or flexography to optimize the production process.

After introducing some fundamental aspects of the inkjet printing process, this review will present an up-to-date overview of the use of inkjet printing in the implementation of different optical elements and photonic devices. In each of the reviewed technologies, main achievements attained, applications and remaining challenges to solve will be discussed.

In particular, a review of the use of inkjet printing in the preparation of microlenses, which are able to couple light in and out optical fibres or to beam shape the light from diode lasers, among other applications, will be presented. Efforts made in the use of inkjet printing in the implementation of waveguides and integrated splitters will also be surveyed.

Special attention will then be devoted to the role of inkjet printing in the field of large area photonic devices, such as organic light emitting diodes (OLED), liquid crystal displays (LCDs) or solar cells. According to the 2015th Roadmap of the Organic and Printed Electronics Association (OE-A), the market for organic and printed electronics was 23–24 billion US$ in 2014 with a predicted increase of 20% in the next years [5]. Development of this application field relies on the effective integration of dielectric, semiconductors and conductive materials over large, flexible substrates leading to thin, light-weight, flexible, even rollable devices with reduced cost and waste of material. Within this large area electronics arena, inkjet printing appears to be a key enabler technology for the cost-effective and high quality manufacturing of the previously mentioned devices with good reliability and yield.

Finally, an overview on the combination of inkjet printing with self-organizing materials, such as colloidal inks containing monodisperse microspheres or liquid crystals, will be presented. The preparation of different photonic devices, mainly comprising periodic structures, and its application as lasers or sensors will also be highlighted.

## 2. The Physics of Inkjet Printing: Drop Formation, Deposition and Fixation

The inkjet printing process essentially consists of the ejection of ink droplets of controlled properties and their precise deposition and fixation on a target substrate [3,6,7,8,9,10].

### 2.1. Drop Formation

**Figure 2:**
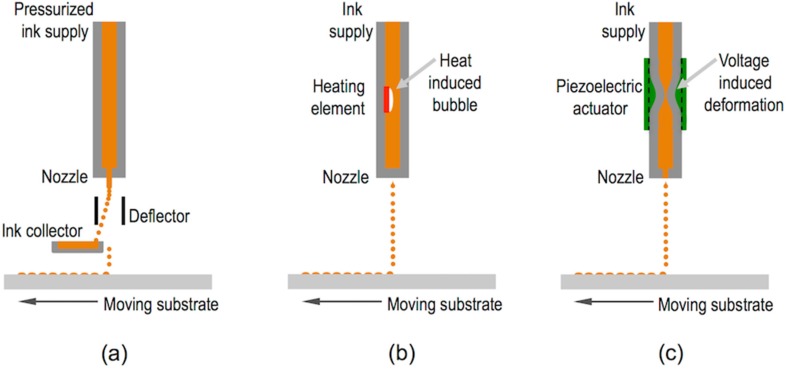
Schematic representation of (**a**) continuous inkjet (CIJ) and Drop on demand (DOD) inkjet printing systems using (**b**) thermal and (**c**) piezoelectric technology.

Two major approaches are usually employed for the generation of ink droplets: Continuous Inkjet (CIJ) printing and Drop on Demand (DOD) inkjet printing (Figure 2). CIJ makes use of the Rayleigh instability to generate a continuous column of droplets that are selectively steered to position them on a target substrate (Figure 2a). Steering is done by the application of a voltage to the ejecting nozzle with respect to ground. When no printing is required the droplets are directed towards a reservoir where the ink is collected and potentially recycled. On the other hand, DOD inkjet printing generates a pressure impulse in the fluid within the printhead able to cause ejection of a droplet at the nozzle that is placed just above the target location of the substrate. Without pressure pulse, the liquid stays at the printhead retained at the nozzle by surface tension forces. The pressure impulse can be generated by the sudden formation and collapse of vapour bubbles at a current induced heating element placed close to the nozzle (Figure 2b). Despite this technology, named Thermal DOD, has become quite popular in many desktop printers, most of the industrial printers and most of the work carried out on functional material printing has been done using piezoelectric technology so this review will mainly focus on this last. This ejection method makes use of a piezoelectric element placed in contact with the fluid cavity (Figure 2c). The voltage applied to the piezoelectric generates a sudden deformation of the fluid cavity and induces a pressure impulse, origin of the ejected drop. With this method, droplets with sizes in the range of the nozzle orifice diameter, typically tens of micrometres, and linear speeds of few metres per second are typically generated at frequencies of 1 to 20 kHz.

**Figure 3:**
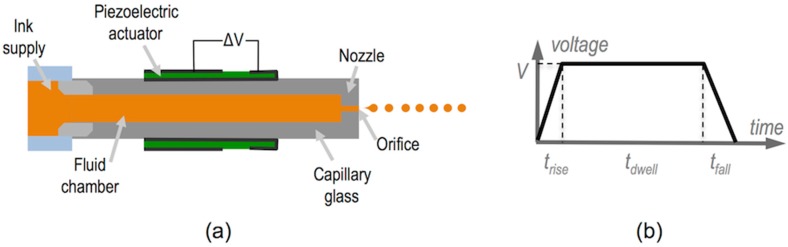
(**a**) Detailed schematic structure of a piezoelectric single nozzle printhead and (**b**) trapezoidal voltage piezoelectric excitation.

As mentioned, for industrial production purposes, printheads have a large number of nozzles, usually hundreds or thousands, that will eject ink droplets. For research purposes, printheads can be as simple as one single nozzle actuated by one single piezoelectric element. Although for practical large area applications single nozzle printheads are not useful and multi-head multi-nozzle inkjet systems are needed, the simplicity of the device helps to understand the underlying physics of the drop formation process so this configuration will be detailed in this section. Figure 3a shows a common fluid tubular cavity geometry consisting of a glass tube with a nozzle and an orifice at one end, and a connection to a supply tube, generally of larger diameter, at the other extreme. The glass capillary has a tubular piezoelectric element bonded around with electrodes in the inner and outer surfaces of the piezo material. In the simplest case, a trapezoidal voltage piezo excitation as the one shown in Figure 3b is used. Initially voltage quickly increases from zero to a voltage V during a short period of time t_rise_. After this, voltage is kept during t_dwell_ and finally it decreases to zero voltage, with t_fall_ being the time needed for this voltage to drop from V to zero.

**Figure 4:**
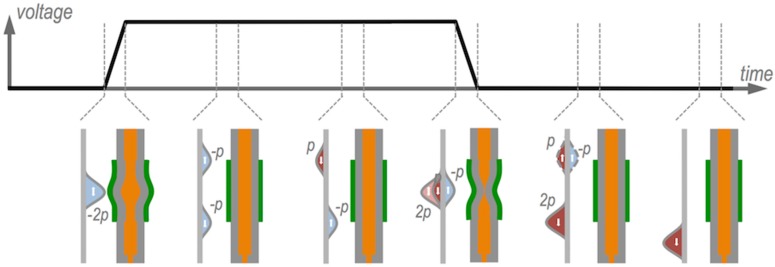
Pressure perturbation generation, propagation and reflection upon trapezoidal voltage application.

The initial voltage rise produces a fast enlargement of the cavity and a subsequent local pressure reduction of the fluid inside. If we assume no dispersion, this negative pressure perturbation can be modelled as splitting into two waves of half the initial amplitude that propagate with no deformation in opposite directions to both ends of the cavity at the speed of acoustic waves propagation in the fluid, as shown in Figure 4. Since the cavity enlarges its diameter at the supply end, we can assume an open-end boundary condition for this part of the cavity with the negative pressure being reflected with a π phase shift turning into a positive pressure pulse of the same amplitude and speed but propagating in opposite direction towards the nozzle end. This nozzle end, on the other hand, can be modelled as a closed-end since the orifice diameter is small compared with the inner diameter of the cavity, so there is no phase shift in the reflection, being the resultant reflected pressure perturbation a negative pressure wave of same amplitude propagating towards the supply end [11,12]. While the voltage is kept during t_dwell_, no new pressure perturbation is introduced in the system. However, the final voltage drop introduces the opposite effect of the initial rise generating a quick decrease of the cylinder radius and therefore a positive pressure perturbation in the fluid that will propagate along the cavity and reflect at the cavity ends following the same described reflection rules at the cavity boundaries. Each time a positive pressure perturbation reaches the orifice end, if sufficient kinetic energy is provided to the fluid to overcome surface tension that retains the fluid within the tube, a column of ink leaves the cavity and a drop can eventually be formed.

It has been experimentally observed that the drop formation process and ejected drop characteristics strongly depend on the pulse characteristics [11,13,14]. Short t_rise_ and t_fall_ times, in the order of few microseconds, are needed to have jet formation. Also voltage needs to be sufficient so the kinetic energy provided to the fluid at the orifice is enough to create new surface in the fluid and to finally generate a droplet. In addition, for a given cavity length, there is an optimum t_dwell_ for which the ejected drop velocity is maximum. This last optimal situation happens when the pulse is such that the final fluid compression (due to the final voltage drop) takes places when the reflected wave originated from the initial fluid expansion (due to initial voltage rise) passes through the piezo location in the middle point of the cavity. As schematically described in Figure 4, the positive pressure perturbation generated at this point cancels the negative pressure wave coming from the orifice end and reinforces the positive pulse coming from the supply end. As a result of this sequence of events only a double amplitude pressure perturbation travels towards the nozzle with no other residual perturbations within the cavity.

As mentioned, the picture described above assumes no energy dissipation of the fluid; however, in real systems, damping due to viscosity, non-ideal reflection and energy losses due to ejected droplets result in amplitude decrease of the pressure waves. The previous model also assumes an initial constant pressure within the cavity that is not the case when ejection of drops is periodically done. In this case, when subsequent pulses are applied, residual pressure waves, originated from previous piezo excitations, travel back and forth between the ends of the cavity. Optimum frequencies can be periodically found if the residual pressure from previous pulses is synchronized with newly created pressure waves being needed, as a result, smaller voltages for drop formation [11,12,15,16,17]. In industrial printers, cavity geometry and voltage actuating pulse are carefully designed to avoid pressure wave reflections reducing in this way this frequency dependence [12].

Besides the acoustics of the printhead and the voltage driven piezo actuation, which confers energy to the leaving column of ink, fluid properties such as surface energy, viscosity and density, strongly influence the process of drop formation after jet ejection. These magnitudes can be combined in a series of nondimensional numbers. While the Reynolds number (Re) relates inertial forces with viscosity (Equation (1)), the Weber number (We) compares kinetic energy with respect to surface energy of the moving fluid when the jet arises at the orifice (Equation (2)):

(1) $$Re=\frac{v\rho a}{\eta }$$

(2) $$We=\frac{v^{2}\rho a}{\gamma }$$

where v is the speed of the droplet, η is the viscosity, γ the surface tension, ρ is the density of the fluid and a the orifice diameter.

**Figure 5:**
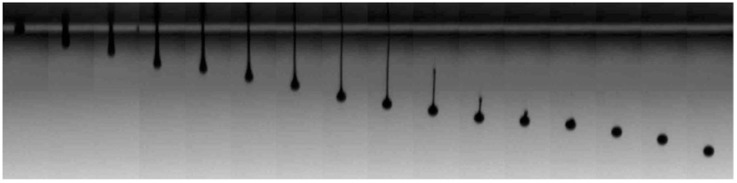
Sequence of photographs showing the drop formation process using a piezoelectric printhead of a hybrid organic-inorganic ink with a small amount of polymeric additive. The time interval between two adjacent frames is 5 µs.

In order to eject well-controlled drops of material, the leaving jet needs to thin and break up after leaving the nozzle (Figure 5). This thinning, which is driven by surface tension forces, is balanced by viscous and inertial forces. For Newtonian liquids with a sufficiently high viscosity, the surface tension that induces jet squeezing is opposed by the viscous stresses within the filament. On the other hand, for liquids of low viscosity, the inertia of the accelerating fluid within the jet is the one opposing the thinning of the jet [18,19].

Wolfgang von Ohnesorge introduced, well before the advent of inkjet printing, a new dimensionless grouping of numbers to understand and define the different regimes found for the jet breaking after it leaves the orifice. The Ohnesorge number Oh (Equation (3)) eliminates the speed of the drop and therefore depends only on the intrinsic physical properties of the fluid and the dimensions of the ejecting orifice (usually similar to the drop diameter):

(3) $$Oh=\frac{\sqrt{We}}{Re}=\frac{\eta }{\sqrt{\gamma \rho a}}$$

The underlying physics behind this number can be better understood by seeing the expression above as the ratio of jet thinning characteristic times of a fluid of high viscosity ($t_{\eta }\sim \eta a/\gamma$), whose thinning is governed by viscous effects, and that of an inviscid liquid ($t_{\rho }\sim \sqrt{\rho a^{3}/\gamma }$), dominated by inertial effects (Equation (4)) [18,20].

(4) $$Oh=\frac{t_{\eta }}{t_{\rho }}=\frac{\eta a/\gamma }{\sqrt{\rho a^{3}/\gamma }}$$

It is known from experimental studies that stable drop ejection typically takes place for certain range of Oh numbers, between 0.1 and 1 according to Reis and co-workers [21]. Slightly narrower range was given by Moon with Oh numbers, between 0.07 and 0.25 [22]. Regardless of the chosen limits, for too large Oh numbers, viscous forces dissipate the pressure perturbation precluding drop formation. On the other side, too low Oh numbers usually lead to columns of fluid leaving the nozzle that finally break into a main drop accompanied by drop satellites.

As said above, the Oh number does not depend on the velocity of the drop, however kinetic energy of the fluid is determinant for a successful drop formation and printing. On one side, a certain threshold of kinetic energy must be transferred to the jet to overcome the surface energy required to generate the droplet. As quantified by Duineveld and co-workers, We numbers should be higher than 4 in order to have ejection (We > 4) [7,23]. On the other side, and related to the landing of the droplet on the substrate, the energy should be low enough to avoid splashing of the fluid when reaching the substrate [24]. This condition has been quantified with the following inequality (Equation (5)) for smooth surfaces [7,24,25]:

(5) $${\left(We\right)}^{1/2}{\left(Re\right)}^{1/4}<50$$

**Figure 6:**
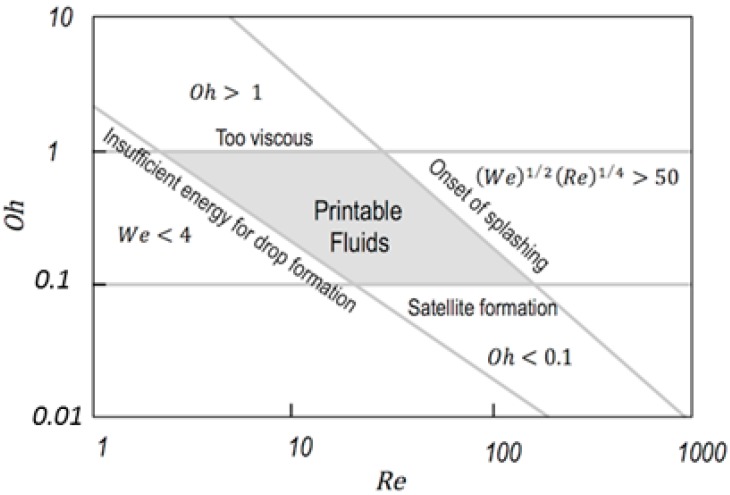
Parameter space of inkjet printable fluids [20].

This set of limits for Oh, We and Re dimesionless numbers allows us to delimitate the properties of fluids that can be printable by using inkjet as schematically presented in Figure 6.

The discussion above has been centred in fluids with Newtonian properties, however it is quite common the inclusion in inks of small amounts of high molecular weight polymers to improve the printability of fluids. In this way, satellites typically formed during jetting of low viscosity fluids can be sometimes suppressed by incorporating viscoelasticity to the ink (see Figure 5). Besides surface tension, viscosity and fluid inertia, the elasticity arising from extensional deformation of the polymer chains within the fluid needs then to be taken into consideration [26,27,28]. Additional dimensionless numbers need to be defined in order to account for all the relevant aspects of the fluid dynamics. For example, the Deborah number (De) establishes the ratio between elastic and inertial forces in a thinning filament, while the Elastocapillary number (Ec) relates the elastic and viscous ones [18,29]. Going back to the previously mentioned example of the addition of traces of polymers to inviscid inks, this makes the De number to increase above certain threshold and turns the material into printable despite the Oh number is not within the established values of printability for Newtonian fluids of Figure 6. As more demanding applications and accurate dispensing of more complex fluids, such as polymeric solutions or colloids with complex rheological properties, are required, the phenomenology and its description in terms of fluid properties becomes increasingly difficult and challenging making the applied field of inkjet printing a challenging playground for basic research of Physics of Fluids.

Concerning the size of the formed drop after filament breaking, it is dictated not only by the fluid ejection conditions but also by intrinsic properties of the fluid. It has been experimentally observed that drop volume increase with decreasing Oh numbers [12], being this fact consistent with the modelling predictions of Fromm [30]. Besides, drop volume can also be controlled by printhead pressure pulse excitation being larger for larger applied voltages [30]. Firing consecutive drops of the same size that fuse together in fly before they reach the substrate is also used to modulate drop volume, a technique that is named greyscale printing. Going back to the voltage excitation pulse, beyond the simple trapezoidal positive one, more complex waveforms, consisting of sequences of positive and negative voltage trapezoidal wave excitations have allowed us to gain control of drop quality, velocity and size. The pinch off of the fluid column from the nozzle can be induced by a properly designed driving waveform generating a negative pressure in the fluid cavity [31]. Fine adjustment of pulse wave parameters allows us to influence the leaving fluid at all the stages of the drop formation process, being also possible a significant reduction of drop diameter with respect to nozzle dimensions [32,33].

### 2.2. Drop Deposition on the Substrate and Ink Fixation

After leaving the nozzle, the droplet flies towards the target substrate. In order to turn flying droplets into a functional device or surface, they need to be precisely positioned on a substrate. During flight, drops lose kinetic energy due to drag of the atmosphere being the positioning of droplets sensitive to air currents that may change the target positioning [23]. For typical inkjet drop sizes and speeds, significant drop speed reduction takes places in distances of the order of few mm. As a consequence, the nozzles should be positioned at distances between 1 and 2 mm to the substrate in order to have precise positioning of the ink on it. This deceleration is more pronounced for smaller size drops and therefore closer distances between nozzle and substrate are required being this an intrinsic limitation of the resolution and printing quality of inkjet technology [34].

**Figure 7:**
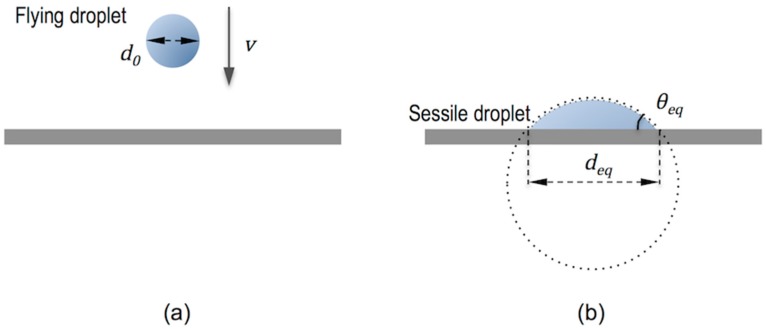
(**a**) Flying and (**b**) sessile droplet dimensions.

The drop finally reaches the target substrate and its behaviour and the underlying mechanism after impact can be again understood by performing nondimensional analysis using We, Re and Oh numbers [35]. For the usual inks and conditions of inkjet printing with droplets of diameter of tens of microns impacting with speeds of few metres per second, gravitational effects can be neglected and splashing is not expected to happen. The drop initially spreads just after impact being this behaviour controlled by inertial forces (impact driven regime). The kinetic energy of the drop is transformed into surface energy by spreading over the dry substrate. After the spreading there is a surface tension driven retraction of the extended drop followed by oscillations of the droplet in which energy is dissipated by viscous forces taking more and more importance capillary forces (capillary driven regime) until the deposited drop reaches the stationary shape that is dictated by surface energy forces [7,36]. As a result, the size of the relaxed deposited drop, and therefore the resolution of inkjet printing technique depends on the size of the ejected drop and the equilibrium contact angle of the ink on the substrate. Since gravitational effects are negligible, the shape of the deposited drop is accurately modelled to that of a spherical cap with base equilibrium diameter $d_{eq}$ given by [37]:

(6) deq=d0(8tanθeq2(3+ (tanθeq2)2))13

being $\theta_{eq}$ the equilibrium contact angle and $d_{0}$ the initial droplet diameter (Figure 7).

High resolution inkjet printheads can produce drops with a volume in the range of one picoliter that corresponds to a diameter of about 10 microns. With a typical equilibrium contact angle of 30°, this flying drop size typically leads to resolutions in the order of 30 microns. As mentioned above, the size can be diminished by adjusting the driving voltage and/or using printheads of smaller nozzle diameter [38]. However, going below 10 microns is problematic not only because clogging is each time much easier to happen but also because such small droplets are quickly decelerated so precise placement becomes more difficult, as mentioned above [23,34].

**Figure 8:**
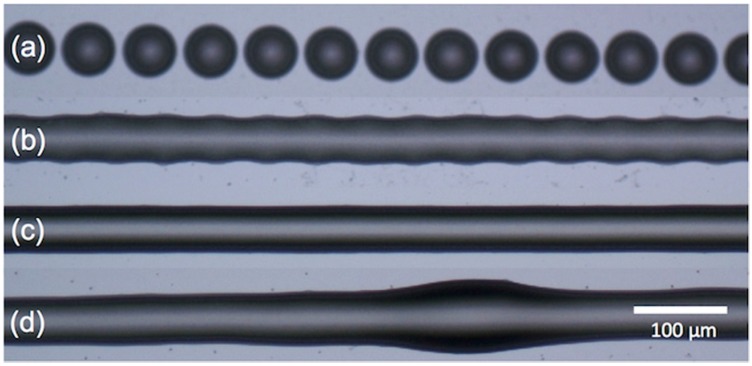
Droplets deposited along a line with decreasing distance between adjacent drops (from **a** to **d**) showing different behaviours: (**a**) isolated dots; (**b**) line with rounded contour; (**c**) line with straight contour and (**d**) line bulging.

Beyond the printing of isolated dots, functional devices need also continuous printed tracks or areas so individual droplets have to be overlapped and the resultant features should be stable to maintain their shape and functionality. When drops of ink are deposited along a line, each overlapping with the previously deposited one, they coalesce into a single liquid bead. If the bead has a freely moving contact line it will be inherently unstable as a jet of water experiences a Rayleigh instability. Conversely, for the case of pinned contact line, the bead can be stable if the contact angle is below 90° [39,40]. Experimental studies carried out by Soltman and coworkers, printing adjacent dots of a water-soluble polyelectrolyte system consisting of poly(3,4-ethylenedioxythiophene) and poly(styrene-sulfonic acid) (PEDOT:PSS), fall within this case since this ink shows zero receding contact angle with the substrate [41]. A rich phenomenology was observed when varying the distance between sequentially ejected drops that is common for many other sets of inks and substrates (Figure 8). For large distances between drops, such that they do not interact on spreading, the individual printed droplets appear as a linear train of dots (Figure 8a). As the distance between printed dots becomes smaller, drop coalescence takes place, forming a line with a rounded contour, reminiscent of the individual contact lines of the original landed droplets (Figure 8b). If drop spacing is further decreased, the undulated contact lines disappear and a transition to parallel side edges is observed (Figure 8c). As mentioned above, for the length scales of inkjet printed drops, gravitational effects can be neglected so the profile of these parallel side edges liquid lines can be modelled in this case as a circular segment determined by the contact angle as done by Stringer and Derby [42,43,44]. If spacing decreases even more, line widening can occur however eventually a bulging instability with regions that outflow the line and other where the line remains uniform might appear (Figure 8d). The appearance of this bulging instability revealed to be deposition rate dependent, as demonstrated by Duineveld [45]. Rapid rate deposition of the liquid in the line produces a local increase of the contact angle that exceeds the advancing contact angle, producing spreading out of the line and therefore a bulge.

Continuous surfaces are also essential features in the preparation of different functional devices such as OLEDs, solar cells or sensors. As in the case of lines, the printed features need to be well defined. Some inks show strong spreading when applied to substrates so the contours of features are not regular or the details are lost. In these cases, the material can be confined by barriers, applied for example photolithographically, limiting the spreading of the ink and therefore defining the contour of the printed features [23,46]. On the other hand, inkjet printing of squared shaped features, with inks showing partial wetting on a flat substrate, has also been studied by several authors. For example, Tekin and coworkers identified that sequential printing of spatially offset dot matrixes covering the whole area can lead to continuous and homogeneous thickness polymeric films if solvents and printing speed are set properly [47]. Seeking also the formation of well-defined rectangular films, Kang, Soltman and coworkers studied different printing schemes. The search of conditions, for example line spacing or line printing sequence, that lead to a liquid bead having a contact angle smaller than the advancing and larger than the receding contact angle is pursued in order to avoid bulging and dewetting respectively, and therefore poor quality in the rectangular film definition [48,49]. These deleterious effects can be overcome by using printing algorithms in which areas are not printed by sequential application of equidistant dots forming lines that later coalesce into areas but sequences of prints heterogeneously distributed in the substrate, a methodology that contributes to have contact angle under control between the limits, therefore keeping well-defined feature shape [49].

Once the ink has been applied, the liquid needs eventually to turn into a solid to effectively implement functional elements and devices. Inks based on waxes, for example, lead to stable features upon a phase transition to the solid state. Inks comprising photopolymerizable monomers can be fixed by its exposure to actinic light. In other cases, inks are polymer solutions or particle dispersions in a solvent. The solvent is, in these last cases, just a carrier for the functional material, and it disappears after evaporation. In this type of inks, the drying process needs to be carefully analysed in order to have the desired quality of the prints.

When a drop of a pure solvent is deposited on a substrate with a smooth surface, evaporation will lead to a continuous reduction of the contact angle until the receding contact angle is reached and the diameter of the deposited drop start to decrease. If the surface is rough the contact line is pinned so that, as evaporation proceeds, the contact angle decreases; however, the droplet does not retract. In case the solvent carries a solid phase, such as a polymer or a small amount of dispersed particles, evaporation of the applied drop, leads to a deposited ring of material with the dimensions of the initial drop. This solid phase at the contact line can prevent the liquid to recede, pinning the droplet to its initial size and controlling in this way the size of the final deposit. However, as evaporation further proceeds in the edge, the loss of liquid in this region can be compensated, as explained by Deegan and coworkers, by flow of liquid from the inner part of the droplet bringing more solid material to the edge where it deposits, what is known as the “coffee stain effect” [50,51,52]. Although this circular ring accumulation of material can be seen as an opportunity for patterning of materials [53,54], usually it has been found as a limitation since it results in poorer device performance [55]. The use of solvent mixtures has demonstrated to be a powerful tool to control the flow of solids and sometimes inhibit the formation of the solid phase ring [47,56]. For example, the use of a mixture of two solvents, one of them having higher boiling point and lower surface tension than the other can lead to a flow that compensates the evaporation induced flow towards the edge described above. The larger evaporation of the highly volatile solvent in the outer part of the deposited droplet generates a compositional gradient in the radial direction and therefore a surface tension gradient. This can generate a Marangoni flow that inhibits the formation of the ring leading in certain cases to a homogeneous deposit [55,57].

## 3. Inkjet Printing for Micro-Optics and Integrated Optics Fabrication

Despite the origins of micro-optics going back a long way (more than two centuries) with the first studies on diffractive gratings by Rittenhouse, later more systematically described by Fraunhofer, it has only been in the last three decades when micro-optics has gained entity and a name as a clear subdiscipline of Optics. Classical passive optical elements such as lenses or mirrors were miniaturized into micro-optical elements, such as microlenses or micromirrors, by the use of well-established photolithographic techniques due to the huge development of the semiconductor industry. Beyond gratings and microlenses, micro-optics has intimately merged with other disciplines of Optics over the last decades. Seeking miniaturisation of optical technologies, micro-optical systems have been integrated together with lasers, LEDs, sensors or fibre communication systems. Nowadays, many optical microsystems rely on combination of micro-optics (usually referring to free-space optical elements) and guided wave optics with the interface between both disciplines blurred. Being at the intersection of many different technologies, advances in micro-optics can broaden the scope of a wide variety of optical microsystems applications [58].

**Figure 9:**
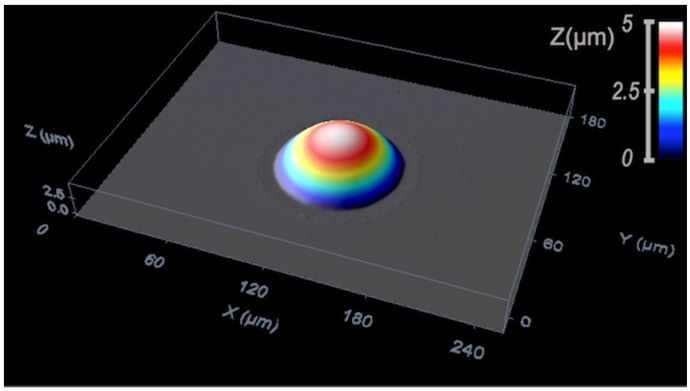
Confocal microscope image of an inkjet printed hybrid organic-inorganic UV-cured microlens deposited on a glass substrate.

Focusing on micro-optics, the Physics to describe classical optical elements, such as cm sized lenses or mirrors, is exactly the same as that used in homologous micro-optical components, with features in the micron range. The effects of diffraction are more important in the latter, or even dominant, as in the case of gratings due to their small feature size in the same length scale of the wavelength of light. After the initial efforts in the field and focus on the preparation of diffractive Fresnel elements through photolithographic techniques, true microlenses were prepared by starting with circular posts obtained with a photoresist, that were subsequently thermally annealed to lead to a spherical cap of molten photoresist, that upon curing results in a lens [59]. Other approaches make use of greyscale photolithographies to obtain polymeric structures that work as refractive microlenses [60]. More recently, inkjet printing has been used in the production of these elements due to the possibility to apply different materials on virtually any type of substrate. Different from photolithographies, inkjet only adds material where it is needed so it allows for the precise application of functional materials at precise locations of prefabricated devices with no or minimal post processing. By using this technology, microlenses with large numerical aperture and short focal lengths can be prepared as an efficient, simple and cost-effective alternative to photolithography (Figure 9).

Final properties of the microlenses can be finely tuned by appropriately selecting the refractive index of the resultant material and by controlling the final shape of the deposited microlens. As mentioned above, for typical sizes of inkjet printing ejected single drops, the sessile drop reaches, after relaxation, a spherical shape that, in the absence of any surface structuring, is mainly dictated by contact angle and the amount of deposited ink (see Equation (6)). This spherical cap shape can be frozen after relaxation for inks based on molten polymers when they are cooled down. MacFarlane and coworkers already reported in 1994 spherical cap convex microlenses made of molten polymer printed by inkjet with diameters ranging between 70 and 150 μm, and focal lengths between 50 and 150 μm [61]. The spherical shape is also kept for prepolymers that are fixed by exposure to UV light after inkjet deposition [62,63].

Other inks contain solvents that need to be eliminated after deposition. This is the case for many studies reported in the literature using SU-8 based inks containing solvents. As in the previously described ink types, wetting plays a very important role in the final properties. Studies carried out printing the same material on substrates with different wetting properties led to microlenses with different geometrical and therefore optical parameters. Besides the wetting, final shape can also be affected by evaporation of ink solvents and subsequent material transfer within the deposited drop [64].

Polymer based solutions have also been used for microlens preparation. Evaporation of solvents in deposited droplets usually leads to large changes in morphology. Pinning of the evaporating droplet fix the radius and induces the flow of the polymer solute within the droplet during evaporation, strongly influencing the final shape of the deposit, usually with a non-spherical profile. However, appropriate selection of the polymer solvent or solvents and target substrate has been demonstrated to lead to an evaporation mode in which the droplet is not pinned and spherical cap polymeric microlenses can be formed [65].

Fabrication of microlenses on substrates with patterned wettability has also been explored to gain control of microlens properties. The preparation of a non-wetting area surrounding the region where the optical material is deposited enables the control of the equilibrium position of the liquid on the surface and therefore the fabrication of several shapes of microlenses. Pre-patterning can be performed by means of photolithographic steps leading to wettability patterned substrates [66]. Pre-patterning can also be assisted by inkjet printing as done by Chen and co-workers [67]. In this last case, during the first patterning step, a solvent (anisole) is deposited by inkjet on a thin layer of PMMA previously casted on top of a flat glass. The solvent acts as an etchant creating, as a result of the coffee stain effect, reproducible well structures with a thinner layer in the centre and a thicker perimeter at the edges. After controlled etching of these patterned polymer layer with O_2_ and CF_4_ plasma, the thinner part of the film is eliminated leaving the bare glass while the thicker rest of the polymer acquires a repellent character. Drops of photocurable optical glue are deposited using again inkjet printing on the generated holes. Self-positioning of the deposited materials is achieved due to the surface tension pattern established during the first step. Fine control of the focal length can be achieved by adjusting the diameter of the wells obtained in the first step and the amount of ink deposited in the second step that is confined by the wells [67].

**Figure 10:**
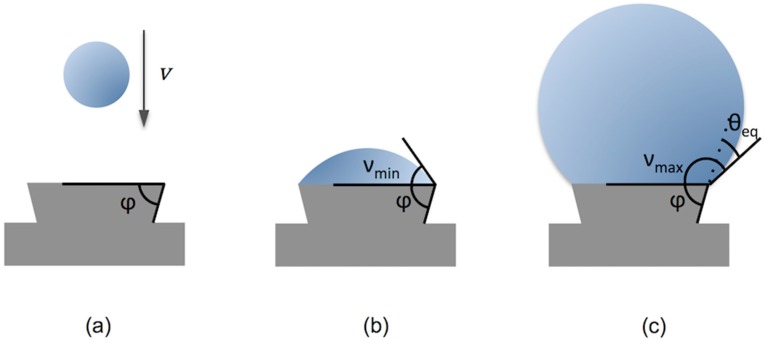
(**a**) Schematic view of a drop being deposited on top of a micro-platform with a rim angle φ; (**b**) The deposited drops are confined by the elevated area fixing the contour shape of the drop. The minimum contact angle $\nu_{min}$ for a drop covering the elevated area is the contact angle $\theta_{eq}$ of the ink on a flat substrate; (**c**) Further addition of droplets leads to an increase of the edge angle until the maximum angle $\nu_{max}$, defined by $\theta_{eq}+\pi -\varphi$, is reached. Above this angle the rounded drop overflows the platform [68].

Topographical features have also been used to control microlens characteristics. Fabrication of large arrays of micro-spherical caps with well-defined height, radius of curvature and edge angles was also performed by pre-patterning substrates with micro-platforms on top of which controlled amounts of an SU-8 based epoxy solution were deposited by inkjet (Figure 10). The deposition of a different number of resin drops in each platform having a well-defined rim angle φ, allows us to gain a precise control of the cap edge angle within a large range between a minimum $\nu_{min}$ defined by the equilibrium contact angle $\theta_{eq}$ and a maximum angle $\nu_{max}$ defined by $\theta_{eq}+\pi -\varphi$. Differences between $\nu_{max}$ and $\nu_{min}$ up to 85° for 100 micron diameter platforms have been achieved by this method [68]. Pre-patterning pillars on substrates has also been used to obtain different lens contour shapes such as circular, elliptical or toroidal [69]. The generation of concave microlenses has also been demonstrated by inkjetting droplets of UV curable prepolymers on photolithographically prepared SU-8 wells [70].

With respect to the materials, besides the molten polymers and typical UV-curable inks, more sophisticated material strategies, seeking enhanced properties such as improved adhesion or higher transparency window, have been investigated. Aegerter and coworkers used sol-gel based inks containing UV curable 3-Methacryloxypropyltrimethoxysilane to make microlenses. They produced plano-convex spherical microlenses transparent from 375 to 2700 nm with diameters between 50 and 300 μm, focal lengths from 70 to 3000 μm and f-numbers (defined as the ratio of the focal length to the diameter of the entrance pupil) as low as 0.61 [71]. Later, the same group went further by printing epoxy-based inks, with similar optical transparency, in the form of plano-convex microlenses of 50 to 1000 μm diameter and focal lengths between 100 and 2200 μm. The smallest separation of the lenses in arrays was approximately 5% of the lens radius and the average surface roughness was as low as 40 nm [72,73].

**Figure 11:**
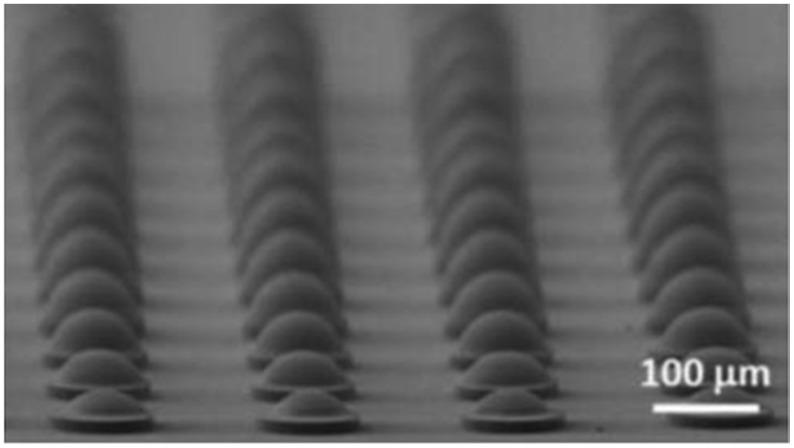
SEM images of microstructured polymeric pillars (diameter 100 µm, height 10 µm) treated with a fluorinated organosilane, with SU-8 microlenses on top formed by inkjet deposition of different numbers of drops (one to ten drops). Reprinted from reference [76] with permission of The Royal Society of Chemistry.

Hybrid organic-inorganic materials were also used to prepare microlenses with high numerical aperture by Brugger and co-workers [62,74]. These lenses achieved a focal distance of 45–50 μm, and a small curvature radius of 29 μm. Periodic arrays of almost semi-spherical microlenses of 50 μm were obtained by treating the substrate with fluorinated silanes. High quality arrays of microlenses with good uniformity and reproducibility, transparency from 400 to 1600 nm and a refractive index of 1.553 at 635 nm were prepared with these materials. By increasing the number of drops per microlens, the focal length increased from 64.1 μm to 175.1 μm and the numerical aperture ranged from 0.41 to 0.30 [75]. By using microstructured SU-8 substrates treated with a fluorinated organosilane, parabolic-shaped microlenses with high numerical aperture, up to 0.86, has also been produced using these same hybrid materials (Figure 11) [76]. Combination of nanoimprinting lithography, to prepare mcirostructured arrays of pillars, and inkjet printing, to create microlenses on top of the pillars, using the same hybrid organic-inorganic material in both structuring techniques, has allowed for the preparation of arrays of microlenses with superior optical performance and high reproducibility. In addition, microlenses with different characteristics, different focal lengths and therefore focal planes have been prepared in the same substrate, separated by just few microns by using this combination of techniques [77].

The typical wavelength application range of microlenses fabricated by inkjet printing has usually been in the visible and near-infrared, however novel materials, as chalcogenides have also been explored to manufacture microlenses for the mid-infrared potentially integrable with quantum cascade lasers in this wavelength range [78]. Fine tuning of lens geometrical parameters with diameters between 10 and 350 μm and estimated focal lengths from 10 to 700 μm were achieved by dispensing small amounts of an As_2_S_3_ solution on a hydrophobic substrate surface and carefully controlling the baking time.

Although, typically all or part of the ink material is intended to remain after the inkjet process, jetting of solvents that eventually completely evaporate has also demonstrated to be a powerful tool in the preparation of microstructured functional surfaces. Inkjet etching using drops of solvents on PS substrates has also been proposed to obtain polymer microstructures which can be used as microlenses [79,80,81]. Depending on the ratio of solvents in the jetted mixture the profile of the surface can be finely tuned going from concave to convex profiles in the same target substrate [80]. Later on, the inverse replica of these microlenses can be obtained by template casting using an elastomeric silicone (Sylgard 184, Dow Corning, Midland, MI, USA) [81].

Microlenses arrays, obtained by inkjet printing, are being explored in different photonic applications. Microlenses have been deposited by inkjet on fibre tips to increase the acceptance angle and more efficiently couple in light from diode lasers. On the other side, microlenses deposited on the fibre tip can collimate the output beam. Inkjet printed microlenses have demonstrated to be a feasible approach to beam-shape the light from edge emitting diode lasers. On the other hand, microlenses precisely positioned on top of arrays of photodetectors can improve their efficiency collecting light [82,83,84,85]. As a result, inkjet is a very attractive technique for the in situ preparation of microlenses for Optical Input/Output (I/O) interconnections for massive high speed switching or parallel processing of information. Integration of different devices such as lasers, photodetectors of fibres has been demonstrated by using inkjet. Microlenses prepared in front of the aperture of a wire-bonded Vertical Cavity Surface Emitting Laser (VCSEL) can help to control the collimation of emitted light and improve the coupling efficiency into fibres being therefore inkjet printing a very effective technology for optical interconnection [86,87]. Recently, Chen and co-workers demonstrated inter-board optical connections between polymeric waveguides using inkjet printed microlenses deposited on top of total internal reflection mirrors. Microlenses of 70 μm in diameter deposited by inkjet printing improved the transmission by 2–4 dB per coupler [88]. Also seeking the coupling out of light from a waveguide, arrays of microlenses with controllable curvature and filling factor, were printed on top of light guide plates by Tseng and coworkers. The prepared systems were able to efficiently extract light with large uniformity (up to 84%) demonstrating the feasibility of this approach for the manufacturing of low-cost LCD backlights [89,90]. Inkjet printed microlenses have also recently been used in the implementation of optofluidic devices for analysis [91].

**Figure 12:**
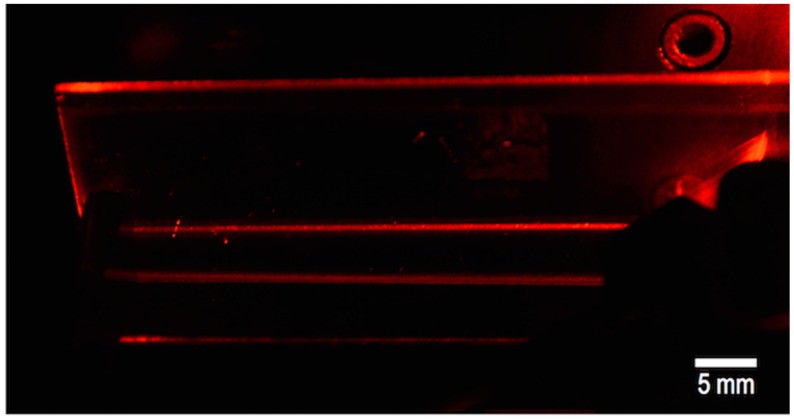
Inkjet printed hybrid organic-inorganic UV-cured planar waveguide deposited on a glass substrate. The 633 nm light propagates from right to left through the waveguide.

Waveguide manufacturing has also been accomplished by using inkjet printing technology. As mentioned in Section 2, printing of lines with parallel ridges can be achieved by sequential printing of closely spaced dots. These lines, that have a spherical cap cross-section, can be used as waveguides if the deposited material has a higher refractive index than the underlying substrate. Planar waveguides can also be obtained by printing larger flat areas (Figure 12). Besides the low cost and rapid fabrication process, an interesting advantage of inkjet printing versus traditional techniques such as photolithography, is the ability of inkjet to directly print high-aspect-ratio complex geometries such as multiple waveguides and splitters taking a simple image file as a design [92,93]. When compared to other printing techniques such as direct writing of cylindrical lightguides using extrusion printing [94], inkjet printing presents pros and cons. While the liquid character of the inkjet printing formulations allows for the preparation of smooth interconnections between optical elements, extrusion printing allows for the manufacturing of free standing optical fibres not achievable by inkjet printing.

The first reference found in the scientific literature about the inkjet printing of waveguides, comes from Microfab Technologies Inc. scientists who reported in 1999 the printing of 100 μm epoxy based waveguides/splitter [92]. The description of these waveguides is detailed in a later publication in 2004, as a 1.74-index material printed on glass with a 1 to 16 splitter with 120 μm wide branches. Edge smoothness of the waveguide-substrate interface was on the order of the wavelength of the transmitted light demonstrating the viability of this technology [95].

In order to make inkjet printing a feasible technology for the preparation of optical waveguides, apart from a precise control of the geometrical parameters, materials with low losses need to be employed. Only few papers after the seminal work of MicroFab have been done in this direction. Chappell and co. employed a commercially available core material (Truemode^®^, a UV-curable acrylate monomer manufactured by Exxelis Ltd., Edinburgh, UK) mixed with solvents to achieve a suitable viscosity to be jetted, and studied the influence of substrate wettability in the deposition process. Despite the authors demonstrate the preparation of lines of core material having a waveguide geometry there is no waveguide optical characterisation reported [96]. SU-8 has also been investigated as a candidate material for the manufacturing of waveguides by inkjet printing; however, loss measurements by Vacirca and co-workers showed high values [97].

Waveguides also constitute a key element in planar optronic sensors for temperature, strain or chemical concentration detection. These are composed of integrated light sources, detectors and sensors, connected by optical waveguides on thin polymer films. A combination of flexographic and inkjet printing technique has also been proposed to prepare these waveguides by using the commercial hybrid organic-inorganic inks InkOrmo as the cladding and InkEpo as core material (Microresist Technology GmbH, Berlin, Germany) [98,99].

To conclude, a recent example has been reported in the preparation of photonic structures using inkjet printing. Patterns were obtained by printing a solvent etchant on a thin film of polystyrene. The solvent dissolves the polymer and this is accumulated at the outer region of the droplet due the coffee stain effect previously described. After solvent evaporation, a microring of typical radius of 50 μm, width of 5 μm and a height of 500 nm is formed. Straight lines were also created following this procedure, acting as waveguides, while microrings including a luminescent dye (1,4-bis(a-cyano-4-diphenylaminostyryl)-2,5-diphenylbenzene) were characterized as lasers showing a quality factor higher than 4 × 10^5^, which is comparable to that of silicon-based resonators [54].

To summarize this section, inkjet printing has been employed in the preparation of microlenses and optical waveguides. A large degree of control of the final properties of microlenses has been achieved through appropriate selection of jetted materials, wetting and topographical features of the receiving substrate as well as post-processing of the deposited materials. On the other hand, the preparation of optical waveguides using inkjet has been much less explored. While photolithographies require complicated or difficult steps to be carried out, sometimes in combination with other technologies, inkjet printing, being a digital, contactless, additive material deposition technique, can be applied as a final step with minimal or no post-processing gaining positions as a relevant technology in the integration of optical micro-devices.

## 4. Inkjet Printing of Light Emitting Devices

**Figure 13:**
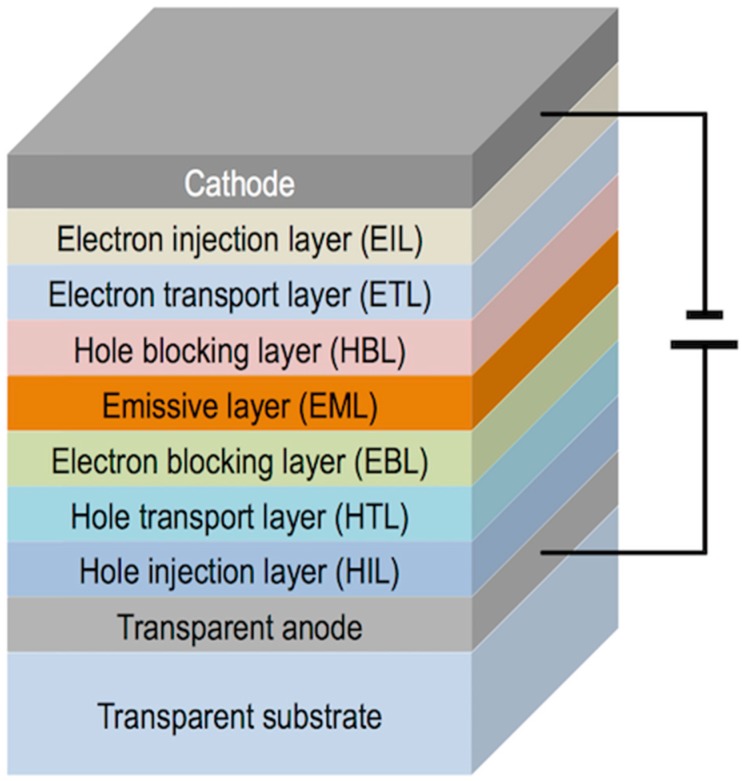
General schematic multilayer structure of an organic light emitting diode.

Organic Light Emitting Diodes are solid-state lighting thin multilayered devices able to transform electricity into light. The structure of OLEDs comprises, in the most simplified version, a thin emissive layer (EL) of an organic emitting material sandwiched between a low work-function cathode (e.g., Ca or Ba) and a transparent anode (e.g., Indium Tin Oxide: ITO) [100]. Additional layers such as hole and electron injection, transport and blocking layers can be integrated in the structure to optimize the transport of charge carriers to the light emissive film (Figure 13) [101]. The inclusion of these layers improves device performance, mainly brightness, threshold voltage, lifetime and efficiency. When a voltage is applied between the electrodes, electrons are injected from the cathode and holes from the anode. Electrons and holes migrate to the emissive layer where they recombine into an exciton that can radiatively decay emitting light. Colour of the emitted light can be tuned through the introduction of changes in the molecular structure of the emitting material. This affects the energy gap of the π–π* transition that dictates the wavelength of the emitted light [102,103].

Currently, OLED emission can be produced with high efficiency, brightness, and uniformity at different colours. OLEDs present excellent contrast ratio (luminance ON/OFF) and fast response time as well as no angle-view dependence. Red, green and blue (RGB) colours can be generated with OLEDs so other colours can be obtained just by combining these three without the need of any colour filters. In addition, the simple thin film structure can be manufactured at room temperature and in some cases from solution processes. All these characteristics, together with the improved lifetime reached over the last years, have made OLEDs systems to already penetrate the market of displays and further improvements in the area can turn this technology in the standard in the flat panel display arena nowadays dominated by LCDs. The ultrathin character of OLEDs allows for the production of flexible devices by preparing them on top of polymeric substrates paving the road for future disruptive display applications. More recently, beyond displays, lighting applications are also gaining a great deal of attention. Besides efficiency, other factors such as durability and intrinsic lightweight, the possibility to combine colours closely matching virtually any power distribution (for example sunlight) with no UV and the ability to adopt complex shapes or bend, make OLEDs a promising approach for future lighting applications [2,5,104,105,106].

Patterning of different emissive materials is a key element in the manufacturing of OLED displays. Vacuum evaporation of small emissive molecules is the prevalent technology to deposit the electroluminescent layers. In combination with masks, this evaporation method is used to manufacture small and medium size displays; however, for large size displays the technical difficulties of this patterning methodology lead to low yields being not competitive with well-established LCD technology. As a result, alternative cost-effective manufacturing methodologies are needed to apply the emitting materials in displays. There is a trend to shift from evaporation techniques to solution based processes for which there is a need of emissive soluble materials. While the solubilisation and wet processing of small emissive molecules have traditionally presented many difficulties, polymeric light emitting materials have excellent solution processability properties being suitable materials for the manufacturing of OLED flat-panel displays [100]. Among the different patterning techniques, inkjet printing has been explored as a solid candidate to produce low cost RGB OLED displays due to its ability to precisely deposit different emissive inks at different positions with minimum waste of material. In addition, it is a scalable technique with reasonably high production speed and can be combined with other R2R processes. Even more inkjet allows us to correct defects in-line increasing with this the reliability and yield of the display production process.

**Figure 14:**
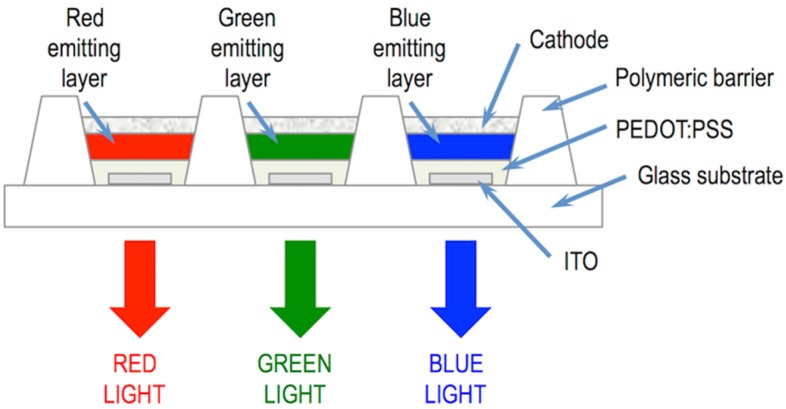
Schematic pixel structure of a red, green and blue (RGB) organic light emitting diodes (OLED) display.

Pixelation of RGB OLEDs by inkjet is done by precisely position droplets of the three colour emissive materials on the substrate [107]. To do this, the receiving substrate provided with the electrodes is patterned with polymer walls surrounding each pixel (Figure 14). The polymer walls are hydrophobic while the substrate is hydrophilic, so when a drop of hydrophilic ink reaches the well, it wets the substrate. If the printhead and the substrate are slightly misaligned or the droplets deviate during flying, these can touch the polymer wall and dewet eventually falling inside the well confining the emissive material. As a result, electroluminescent pixels with position and size accuracy within one micrometre, required for this technology, can be processed. High resolutions exceeding 200 pixels per inch, that are suitable for large size but also for medium and small size displays can be created using this approach [108].

The first attempts to incorporate inkjet printing technology in the production of OLEDs were disclosed at the end of last century. Hebner and co-workers prepared patterned emitting devices by inkjet printing three different chloroform solutions of polyvinylcarbazol as a hole transport polymer doped respectively with coumarin 6 (C6), coumarin 47 (C47), and nile red as light emitting chromophores. Low threshold voltage and luminescence similar to that obtained by spin-coated devices was found for these processed with inkjet [109].

Almost simultaneously, the group of Yang at the University of California developed different configurations of OLED devices [110]. In a first example, they inkjet printed a solution of PEDOT on an ITO substrate. After this PEDOT patterning, a layer of Poly[2-methoxy-5-(2′-ethylhexyloxyl)-1,4-phenylenevinylene] (MEH-PPV) was applied by spin-coating and finally a Ca cathode was deposited. The differences in conductivity between the PEDOT patterned areas and the non-printed regions led to differences in emission, allowing for the definition of logos and images [111]. The same group developed new different devices using inkjet technology. They built a dual colour polymer based emitting pixels by initially applying by spin-coating on an ITO substrate a blue emitting water soluble polymer, poly[2,5-bis[2-(*N,N,N*-triethylammonium)ethoxy]-1,4-phenylene-alt-1,4-phenylene]dibromide (PPP-Net+3). On top of it, a red-orange emitting, water soluble polymer, poly(5-methoxy-(2-propanoxysulfonide)-1,4-phenylene vinylene) (MPS-PPV), was patterned as pixels by inkjet printing. The inkjet patterned ink partially diffuses into the underlying layer. After the evaporation of the cathode the film presented blue emitting areas where no red ink was deposited whereas the printed areas showed the same red-orange electroluminescent spectrum of the MPS-PPV that was ascribed to the dopant diffusion and energy transfer effects [112]. A more sophisticated version of a multicolour RGB OLED was prepared by the same group. To implement it, a blue emitting polymer, poly-9-vinylcarbazole (PVK), was applied by spin-coating on top of an ITO glass substrate. Similar as in the previous case, two different methanol solutions of tris(4-methyl-8-quinolinolato)Al(III) (Almq3) and (4-(dicyano-methylene)-2-methyl-6-(4-dimethylaminostyryl)-4*H*-pyran) (DCM) were inkjet-printed on the PVK layer. After the elimination of the solvents and the deposition of the cathodes, the application of voltage led to blue emission in the PVK area, orange-red emission in the PVK/DCM regions and green-blue emission in the PVK/Almq3 ones [113].

Beyond these initial attempts, Kobayashi and coworkers incorporated a thin film transistor (TFT) array in the substrate, as the one used in LCDs, to individually address the deposited RGB pixels. Despite only red and green pixels were deposited by inkjet printing, this work was a clear precursor for the preparation of active matrix OLED (AMOLED) displays by inkjet printing technology [114,115].

The effective incorporation of inkjet to OLED display manufacturing requires efforts and developments in different aspects of the inkjet process that comprehend the material to be deposited, the ink formulations, their deposition and drying process, the post-processing after inkjet as well as the equipment to pattern the large area devices.

**Figure 15:**
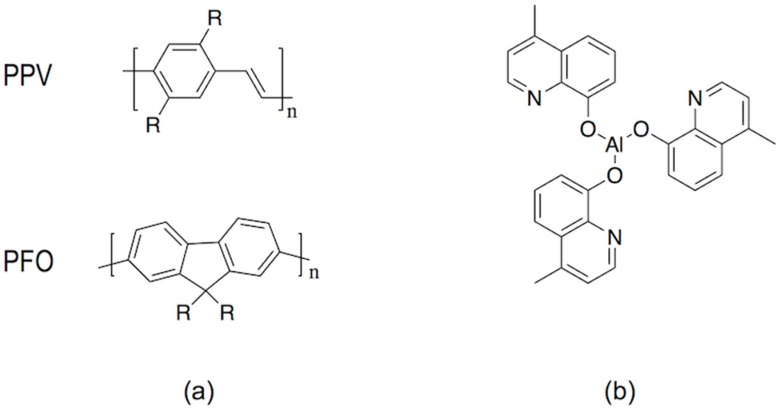
Chemical structure of materials used in OLEDs preparation: (**a**) light emitting polymers poly(phenylene vinylene) (PPV), polyfluorenes (PFO) and (**b**) light emitting small molecule Tris(4-methyl-8-hydroxyquinoline)aluminum.

As in the case of other solution processes, the deposited materials by inkjet need to have the appropriate optical and electrical properties to fulfil the stringent demands of display applications. Focusing in the electroluminescent materials, light emitting polymers such as poly(phenylene vinylene) (PPV) derivatives, MEH-PPV for example, which are soluble in common organic solvents, have been used in the preparation of OLEDs by inkjet printing [114,116]. The lifetime of the emitting materials and the device itself has been crucial in the effective incorporation of OLED technology in real applications, especially critical for blue OLEDs [101,117]. New macromolecular architectures, such as polyfluorenes and poly(spirofluorene)s, have led to solution processable materials with increased lifetime suitable for inkjet fabrication of OLEDs (Figure 15) [114,116,118]. Purification of these polymeric materials is a key element to make lifetime longer in OLED materials. When compared to polymers, higher levels of purity can be achieved in small emissive molecules, typically used in the preparation of OLEDs by evaporation, however these cannot easily be processed from solution as already mentioned. As a result an intense effort has been made towards the chemical modification of these molecules to make them soluble and inkjet-processable [119,120] and also towards the manufacturing of devices using them [121,122]. Exposure to moisture and oxygen results in degradation of the performance and lifetime reduction so the encapsulation of devices after printing is nowadays a standard step in the manufacturing of OLEDs [123,124].

**Figure 16:**
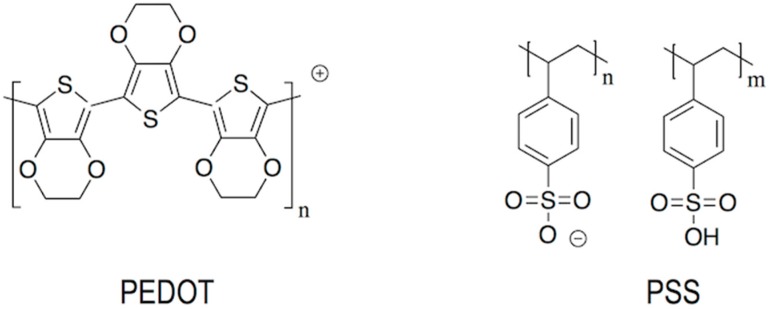
Chemical structure of poly(3,4-ethylenedioxythiophene) and poly(styrene-sulfonic acid) (PEDOT:PSS) polyelectrolyte commonly used in OLEDs preparation.

PEDOT:PSS has been widely employed as an organic material to planarize the ITO electrode and to facilitate the transport of holes towards the electroluminescent layer (Figure 16) [125]. Inks of this polyelectrolyte system have been prepared and processed by using inkjet for the manufacturing of OLEDs [116,126,127,128]. Control of the PEDOT:PSS ratio, the solvents employed or the addition of surfactants to inks, strongly influence the morphology and the electric properties of the final applied materials after solvent evaporation [129]. Due to the excellent optical and electrical properties of PEDOT:PSS, this is ubiquitous in OLED literature usually in combination with the ITO layer. However, motivated by the scarcity of Indium, it has been also tested in the manufacturing, also using inkjet, of devices with no ITO layer being PEDOT:PSS the anode material [128,130,131,132].

Besides suitable deposited materials properties, inkjet processing imposes additional demands with respect to conventional solution based processes. Functional inks must fulfil the requirements of rheology and surface tension to be successfully ejected [126]. They usually comprise a solvent and the functional materials, with a typical solid content between 0.2% and 2.5% w/v, leading to a solid film in the order of 100 nanometres after solvent evaporation [133]. These solvents need to be carefully selected to facilitate ejection without nozzle clogging. In addition, being OLEDs multilayer structures, the solvents used should not attack previously deposited layers. Crosslinking of the applied layers is a commonly used strategy to avoid damage caused by subsequently deposited materials [134]. Recently, Coenen, Gorter and co-workers at the Holst Centre in Eindhoven, demonstrated the fabrication of OLEDs with three adjacent organic functional layers [121,122]. In one of the examples disclosed by this group, a PEDOT:PSS layer, an additional thermally crosslinkable hole injection thin film and an emissive layer were successfully stacked by using inkjet printing [122].

Although the general trend is that the deposited material should not affect the properties of the underlying layer, inkjet can also be employed to locally induce chemical reactions in the substrate through the so called Reactive Inkjet Printing (RIP) technique. In the field of OLEDs, the group of Jabbour used this technique to locally modify the resistivity of PEDOT:PSS. The deposition using inkjet printing of a hydrogen-peroxide-based ink on top of the conductive layer produces oxidation and an increment of the sheet resistivity. This local modification of electrical properties allows for the preparation of logo and greyscale systems by using this simple approach [130,135].

The properties of the applied film after drying, especially morphology, thickness and its uniformity, must be also carefully controlled since they strongly influence electrical current density and emission properties of the deposited layer. As mentioned in Section 2, solvent evaporation can lead to pinning and coffee stain effect. This effect poses a challenge in OLED manufacturing since it results in inhomogeneous film thickness with detrimental consequences in OLED performance. The proper selection of solvents or mixtures of them have demonstrated to be key elements for the preparation of uniform thickness devices [56,57,136]. Ink concentration, substrate temperature and inkjet printing dot spacing also largely affects the evaporation process as already described in this review. Control of all these variables has allowed for the preparation of uniform thickness, good quality deposits for OLED applications [137].

Besides being a tool for device micromanufacturing, inkjet printing has demonstrated to be a valuable tool for material and process evaluation and optimisation since it can generate batteries of samples processed with different conditions in a very reproducible fashion [56,138,139,140,141,142]. For instance, Tekin and co-workers prepared thin film libraries of six alkoxy-substituted poly(p-phenylene-ethynylene)-alt-poly(p-phenylene-vinylene)s (PPE–PPVs) with a systematic variation of thickness. The evaluation of the optical properties of the different inkjetted films allowed us to identify the influence of alkoxy side chain substituents and film thickness in the luminescence properties of the materials [139]. Studies on the printability of MEH-PPV solutions of different concentrations and solvents via inkjet were also carried out by the Schubert group. The morphology and surface quality of the obtained films was studied as a function of printing parameters [140]. The influence of other variables such as ink composition, dot spacing and the substrate temperature has also been explored in poly-(phenylene-ethynylene)-poly(phenylene-vinylene)s copolymer systems by evaluating thin film libraries prepared by inkjet printing. Fast parameter screening allows us to identify adequate conditions to obtain homogeneous films of fixed thickness being therefore a valuable tool for device manufacturing [141]. Relevant relationships between ink viscosity, polymer molecular weight, and film thickness and roughness have also been established by using this combinatorial approach [142].

Another shortcoming to be addressed in OLED technology has been the lack of pure colours due to the broad emission of organic materials, usually showing a full-width half-maximum (FWHM) of around 70 to 100 nm. To overcome this issue quantum dots (QDs) have attracted a great deal of attention since they present highly efficient narrow band emissions (FWHM of 20 to 30 nm) whose wavelength emission peak can be tuned simply by changing the size of the nanoparticle. QDs can be in situ prepared by Reactive Inkjet Printing as demonstrated by Song and co-workers. In this case, the deposition of a polymer and a cadmium precursor done by inkjet printing, followed by a treatment with hydrogen sulphide gas, led to CdS QD polymeric composites [143]. However more common approaches to synthesize QDs are carried out in solution. Once prepared, they are surface-modified to make them compatible with common solvents allowing for the processing from solution. This enables the preparation of QD light emitting diodes (QDLEDs) that present high colour purity leading to improved colour saturation and colour gamut with respect to conventional OLED displays prepared using organic molecules [144,145,146]. As a remarkable example, Samsung scientists have demonstrated the patterning of QDs and the preparation of QD displays by transfer printing using an elastomeric stamp. Although this step is a major breakthrough, handling of large area stamps, needed for large area display manufacturing, is not trivial and stamps can be damaged due to repeated use resulting in a loss of printing quality [147]. As for other solution processable materials, inkjet printing appears as a suitable candidate for the preparation of QD displays.

**Figure 17:**
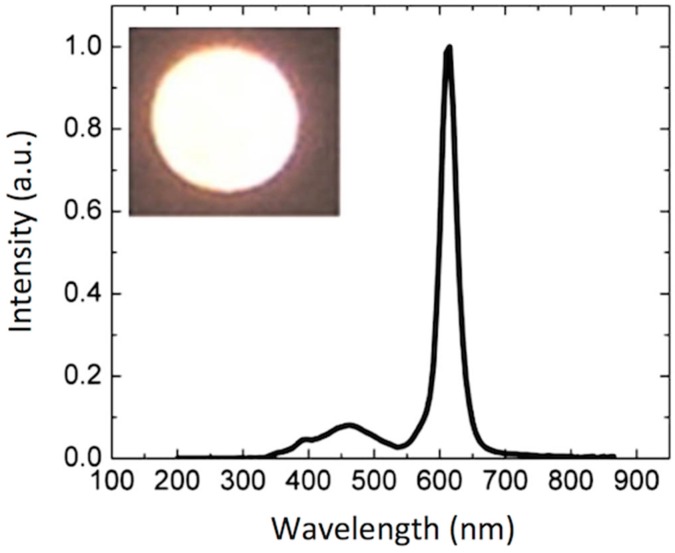
Electroluminescence of a QDLED showing emission from the quantum dots (sharp peak near 600 nm). A real operating device with an area of 0.14 cm^2^ is shown in the top-left part of the plot. Adapted from [148], with the permission of AIP Publishing.

The inkjet printing of QD formulations consisting of a CdSe core and a CdS/ZnS double shell dissolved in chlorobenzene was demonstrated by Jabbour and co-workers. QD single pixel light emitting devices were prepared by inkjet printing a layer of QDs as the emissive layer embedded between a spin-coated hole transport polymer and a thermally evaporated organic molecule electron transport layer (Figure 17). Quarter video graphics array (QVGA) monochrome displays (320 × 240 pixels) were built using inkjet printing of these materials [148]. The group of Bulovic at MIT demonstrated the inkjet printing of QD—polymer composites leading to narrow band emissions in the red and in the green region [149]. RGB QD AC-driven displays were prepared by using a layer of a blue electroluminescent emitting layer of a commercial phosphor powder that constitutes the blue pixels and illuminates QDs that down-convert blue light into red a green depending on the pixel. Jabbour and co-workers also demonstrated patterning by inkjet printing technology of QVGA displays with QDs inks emitting in the red, green and blue [150]. Besides QDs, fluorescent inks based on semiconductor nanorods have also demonstrated to lead to highly concentrated printed layers with little emission shift and high quantum emission yield [151].

Large format (60 inch), Ultra High Definition (4K UHD) format are being attempted to be inkjet printed with QDs inks [152]. On the other hand, efficient (Quantum efficiencies larger than 10% for all three RGB colours), long lifetime (300,000 h) and well controlled RGB peak wavelengths potentially leading to a colour gamut largely exceeding current colour standards for television, has been achieved using QDs solution processable materials by researchers from Nanophotonica Inc. and the University of Florida [153,154]. These studies demonstrate that inkjet printing of this type of material represents an extremely attractive approach for the manufacturing of ultra high definition-large format displays.

Beyond the materials, in which large chemical companies as Merck KGaA are heavily involved [120,155], a large effort is also being devoted to optimize the industrial manufacturing process. For example, DuPont Displays is trying to simplify the production process removing the polymer walls surrounding each pixel and therefore eliminating one photolithographic step reducing therefore cost production. To do this they have developed the nozzle printing technique in which a continuous liquid jet moves across the substrate aligned with previously defined wetting and non-wetting areas. As a result, continuous lines of emissive material instead of aligned pixels are obtained [156].

Focusing on the manufacturing equipment, the company Kateeva, that counts with the financial support from Samsung, has developed an inkjet printing platform for the production of flexible and large scale OLEDs. This equipment produces in a dust-free, nitrogen environment, avoiding oxygen and moisture leading to improved device lifetime. In addition, through process-control monitoring and printhead control algorithms, visual heterogeneities in the printing, referred to as mura, due to nozzle to nozzle non uniformities, are strongly reduced [157].

Summarizing this section, OLED technology has tremendously improved over the last two decades. Although small OLED displays can be routinely manufactured by evaporation processes, production of large area displays present still many challenges. Nowadays, solution-processable materials present excellent performance and equipment for OLED inkjet printing with reasonably high throughput, reliability and yield is being developed. With all this background it can be stated that inkjet printing is well positioned to become a key enabling technology to manufacture future large format, high-resolution displays.

## 5. Inkjet Printing of Other Flat Panel Displays Beyond OLEDs

Advantageous aspects of inkjet printing for OLED display manufacturing such as the digital character of the technique, its scalability, the low waste of material and the possibility to combine with R2R production techniques for flexible devices are also of great relevance in the production of other types of displays such as LCDs or electrophoretic displays (EPD) [158]. As a result, inkjet has also been explored as a valuable tool in other flat panel display technologies.

**Figure 18:**
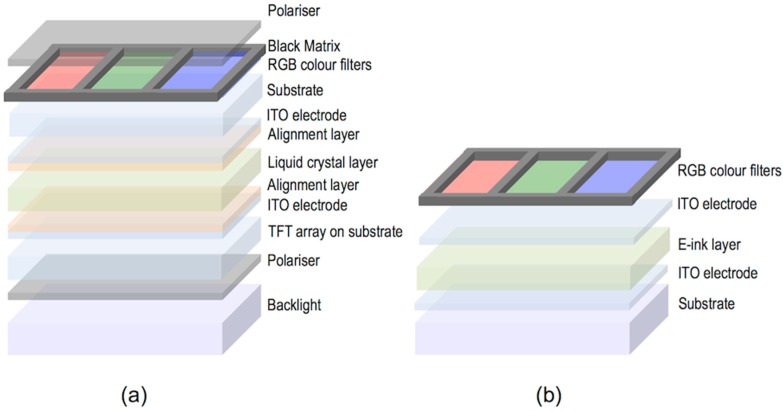
Schematic multilayer structure (**a**) an RGB liquid crystal display (LCD) and (**b**) a RGB e-ink display.

LCDs, the dominant technology nowadays in flat panel displays, are, as OLEDs displays, multilayer structures able to deliver digital visual information to users. High-resolution full-colour images are nowadays a standard in LCD technology; however, the search of new approaches to reduce costs in a mature market is an always-present target for the LCD industry. A LCD typically consists of two thin transparent substrates. These substrates are separated few micrometres having electrodes and alignment layers in the inner part of the assembly. The gap between the glass plates is filled with a liquid crystal (LC) whose orientation in the absence on an electric field is controlled by the alignment layers. The application of an electric field changes the orientation of the LC and therefore the polarisation state of the light passing through the LC layer. This electrically controlled change of light polarisation in conjunction with properly oriented thin film polarisers allows for the control of the transmission at each individual pixel. Differently from OLEDs, pixels in LCDs are not self-emitting, and therefore a backlight is required for illumination. To generate colour images each pixel is subdivided into three independent adjacent subpixels having RGB colour filters. An absorbing structure, named Black Matrix, is placed in the area between the colour filters preventing light leakage between subpixels and acting as a light shield for TFT elements. Figure 18a shows a simplified version of a typical LCD. Electrophoretic displays, based on the spatial reorganisation of pigment particles under the action of an electric field, can also be manufactured having a similar RGB colour filter architecture as shown in Figure 18b.

The fabrication of the colour filters, currently done in the LCD industry through numerous complex photolithographic steps, takes up to 25% of the overall material cost of the display [159]. A way to reduce costs in display manufacturing is to replace photolithography by other printing technologies, with inkjet printing being especially appealing. Besides the advantages mentioned at the beginning of this section, the number of steps for patterning is drastically reduced with respect of photolithography potentially reducing production costs. Efforts in developing inkjet printing technology and deposition strategies have been done to pattern colour filters in LCD panels [160,161,162]. Many of the challenges and difficulties to print colour filters are common to those found in the printing of light emitting materials in OLED display manufacturing so solutions will also be common or at least parallel. As an example, the non-uniformity of the colourant film thickness leads to undesired visual effects, so accurate control of drop volume and positioning are crucial. Colour filters for LCDs can be applied into a pixel size wall structure pre-patterned on the top substrate of the LCDs similar to that typically used in OLED manufacturing. Actually, the black matrix can perform this function by acting as a barrier that precludes ink to mix with that of adjacent pixels. Although this black matrix structure is currently prepared by photolithographic techniques, its preparation using inkjet technology has also been reported [163].

Wettability and therefore treatments of the pre-patterned substrate are, as in OLED manufacturing, a determinant for the quality of the printed elements [164]. The group of Chang and co-workers showed that CF_4_ plasma treatment of the substrate prior to printing leads to improved colour uniformity of inkjet printed colour filters when compared to O_2_ plasma treatment [165,166].

Efforts have also been made in the study of formulations, usually based on inorganic pigments or dyes, towards the improvement of colour characteristics, ink stability and printability. Additives such as hyperbranched polymers have been added to tune the ink rheology and therefore its printability [167]. Diblock copolymers have been included too as dispersants in ink pigmented formulations leading to improvements of the mechanical properties and chemical resistance of printed colour filters [168]. Improvements in transmittance, colour purity as well as spectral and thermal stability have been sought by the inclusion of nanoparticles [169,170] or specifically designed dyes such as perylenes and phthalocyanines [171].

Although the research carried out in the inkjet printing of colour filters for displays has demonstrated that is a feasible technology, it still presents some challenges to be effectively introduced in the display manufacturing industry. As mentioned, some of them, such as the control over the manufacturing process, control of droplet volume of different nozzles or jetting failure, are common to those found in OLED displays manufacturing. Solutions for these issues, relying on the development of process-controlled production equipment in a clean and controlled environment could make inkjet printing technology to be an effective part in the industrial display mass production.

**Figure 19:**
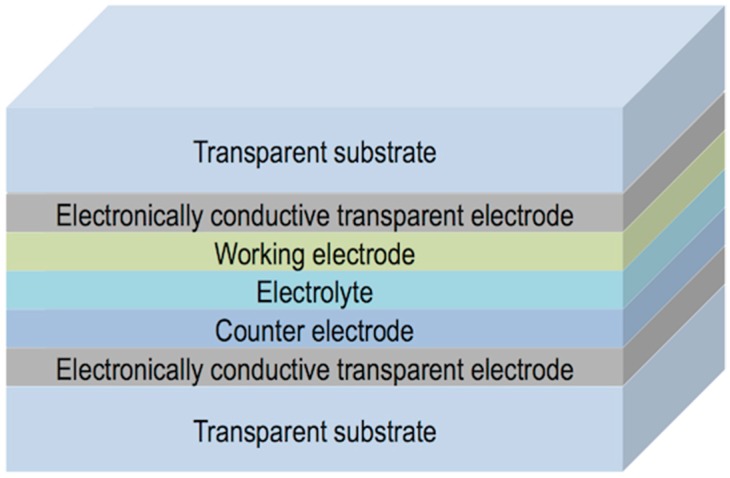
Schematic multilayer structure of an electrochromic device.

Finally, inkjet printing has also been explored by some groups as a tool for the preparation of electrochromic devices. A simplified version of this device, as the one shown in Figure 19, is quite similar to an electrochemical cell consisting of two conductive substrates (at least one of them being transparent to let the light go through). In one of them there is a layer of electrochromic material as a working electrode while in the other it is deposited a counter electrode. Between the two layers there is an electrolyte [172,173,174]. When a small voltage is applied, an electrochemically induced oxidation–reduction reaction takes places leading to changes in the absorption bands of the material.

Wallace, in het Panhuis and coworkers disclosed water based jettable formulations containing polyaniline and a large fraction (up to 32%) of functionalized multi-wall carbon nanotubes. Printed films of these inks on platinized indium tin oxide coated glass and gold coated poly(vinylidene fluoride) substrates displayed polyelectrochromic behaviour switching between yellow, green and blue [175].

Tungsten oxide nanoparticles have also been used for the preparation of electrochromic inks [176,177,178]. As an example, Laia and co-workers prepared water based formulations containing these nanoparticles that were inkjet printed on flexible ITO-coated polyethylene terephthalate (PET) substrates to form both, the working electrode and the counter electrode having an electrolyte in between. The application of an external voltage led to the formation of different images demonstrating the possibility of displaying information in these inkjet printed devices [176]. Lee, Magdassi and co-workers recently developed, besides tungsten oxide inks, nickel oxide formulations to prepare electrochromic devices by inkjet printing. By using layers of these two materials as complementary electrodes, a large optical modulation of 75% at 633 nm was achieved, attributed by the authors to the synergistic and complementary electrochromic behaviour of the two printed layers [179].

**Figure 20:**
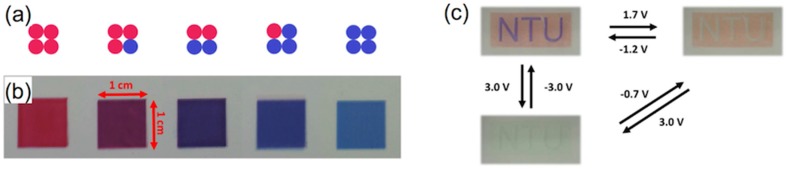
(**a**) Colour-mixing patterns resulting in (**b**) electrochromic material thin films with different mixing ratios of red and blue; (**c**) Multicolour patterns and colour states under different applied potentials. Adapted with permission from reference [180]. Copyright (2015) American Chemical Society.

Multicolour electrochromic devices have also been inkjet printed by using solutions of two metallo-supramolecular polymers, derived from iron and ruthenium, presenting two primary colours, blue and red respectively. Besides the formation of bicolour images, digital control of the deposition of the two inks in dot arrays with different red and blue dot ratio allows us to create areas with various colours without the need of premixing or new material synthesis (Figure 20) [180]. More recently, the group of Reynolds at the Georgia Institute of Technology has developed Cyan/Magenta/Yellow to colourless electrochromic inks based on alkoxy-functionalized poly(3,4-propylenedioxythiophene)-based polymers. By appropriate application of the inks, either layered or patterned over the conducting electrode, a broad range of colours can be achieved while keeping fast switching and high contrast. This makes inkjet printing a robust methodology for the preparation of patterned colourful electrochomic devices for the displaying of information [181].

## 6. Inkjet Printing of Organic Photovoltaics

The Sun provides Earth with 174 petawatts (PW) of solar radiation being by far the most abundant energetic resource on Earth [182]. Nevertheless, photovoltaic energy is still after hydro and wind energy in terms of global installed capacity with a total of at least 227 GW by the end of 2015 [183]. Among the existing photovoltaic technologies available, highly efficient crystalline silicon solar cells, which have reached a mature state, currently dominates the market, however the elevated costs of the materials employed and their processing, limits a widespread integration of photovoltaics in the Energy landscape. Although attempts to reduce costs in silicon solar cell manufacturing have been undertaken, for example, by substituting some of the photolithographic or spatially selective doping steps by inkjet printing based processes [184,185], there is a need of major breakthroughs to put photovoltaics in a prevalent position in the Renewable Energy field.

**Figure 21:**
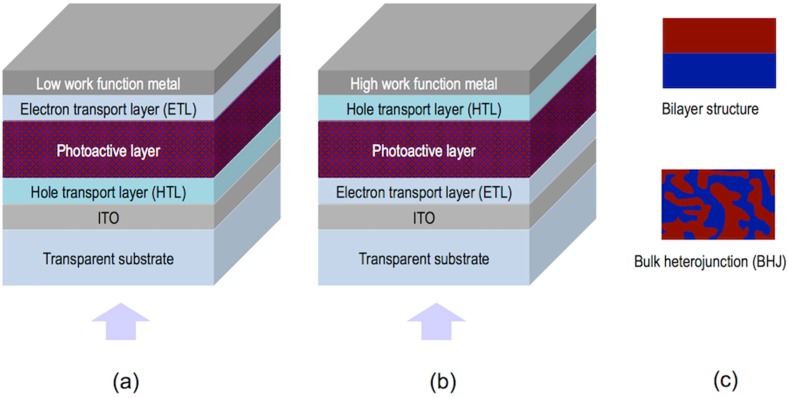
Schematic layer structure of a (**a**) conventional and an (**b**) inverted OPV device; (**c**) The photoactive layer, comprising electron donor and acceptor materials can have a bilayer or bulk heterojunction structure.

As an alternative to silicon photovoltaics, the use of solution processable conjugated polymers that strongly absorb light in the UV-Vis-NIR region has been largely recognized as a viable route towards cheaper and sustainable large-scale photovoltaic modules [186]. The most common organic photovoltaic device structure comprises an anode, a photoactive layer and a cathode with at least one of the electrodes being transparent to allow the light to reach the photoactive material. As in the case of OLEDs, additional layers are usually introduced between the electrodes and the photoactive layer, in this case of solar cells to facilitate charge extraction from the photoactive layer. An electron transport layer is usually placed at the cathode side while a hole transport layer is placed close to the anode. A differentiation is done depending on the location of the cathode with respect to the transparent substrate. If the cathode is away from the substrate, the solar cell architecture is termed as conventional (Figure 21a) while the solar cell is named inverted if the cathode is on the substrate (Figure 21b).

Concentrating on the photoactive layer, bulk heterojunction (BHJ) solar cells comprising an electron donor conjugated polymer and an electron acceptor fullerene based material, have received great attention as one of the most promising approaches to produce low-cost solar energy. Compared to bilayer architectures in which the photoactive layer comprises two separate films of the electron donor and acceptor materials with well-defined interface, bulk heterojunctions are based on a blend of the two systems (Figure 21c). The phases of the two components are interpenetrated forming a bicontinuous heterojunction. Absorption of light generates bound electron–hole pairs or excitons in the electron donor material that can diffuse, before recombination, to the interface of the two materials and dissociate to generate free charge carriers. The large interfacial area of the heterojunction with typical length scales below the exciton diffusion length, improves the efficiency of exciton dissociation since it diminishes the exciton travel distance. Therefore, control of the morphology of the heterojunction is crucial to optimize device performance. On one hand, exciton dissociation at the interface of the materials needs to be maximized and on the other hand charge carrier extraction to the electrodes must be as efficient as possible through the donor and acceptor continuous channels of the two materials [187]. Efficiencies larger than 10% have already been achieved with this type of BHJ solar cells [188]. Besides these single photoactive layer devices, multijunction solar cells, so called tandem solar cells, can be created by judiciously combining several junctions in one single solar cell. The complementary absorption of the different layers broadens the effective absorption window of the stack covering a larger part of the solar spectrum further increasing the power conversion efficiency [189,190].

Although important milestones have been reached and improvements are still in progress in terms of power conversion efficiency of organic photovoltaic (OPV) solar cells, their stability needs to be concurrently improved. Also, low-cost and high-throughput production processes need to be developed to make this technology successful and to penetrate the market.

As in the case of OLEDs, materials used in OPV can be processed at low temperature from solution and scaled up by using R2R technologies. In particular inkjet printing of OPV materials has drawn a lot of attention. Although inkjet has lower throughput when compared to other well established printing techniques such as screen printing, inkjet is a noncontact, digital technology able to print a wide variety of materials as layers, even several of them in the same device, and also apply conductive tracks needed to interconnect photovoltaic modules. All this makes inkjet a very attractive technology for the large-scale production of low-cost photovoltaic modules [191,192,193,194,195,196,197].

**Figure 22:**
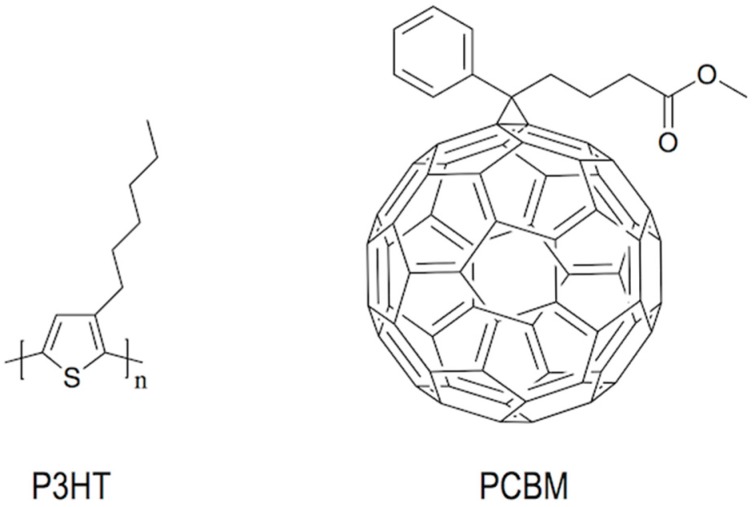
Materials such as poly(3-hexylthiophene) (P3HT) in its regioregular form and 1-(3-methoxycarbonyl)-propyl-1-phenyl-(6,6)C61 (PCBM) typically used in the preparation of BHJ.

Research efforts have been done in the inkjet printing of the different layers of the device focusing especially in the photoactive materials (Figure 22). For example, Schubert´s group has undertaken the preparation by inkjet printing of thin-film libraries of different polymer:fullerene materials for potential application in BHJ solar cells. They systematically prepared libraries of materials with a variety of blend ratios and solid concentrations, different solvent ratios, and film thicknesses and later on performed combinatorial screening of the optical and morphological properties of the films. Beyond fundamental knowledge acquired in these particular studies, these works present a fast and powerful experimental approach to establish processing-structure-properties relationships in OPV materials of crucial importance for the preparation of solar cells [198,199,200].

Several studies can be found in the literature on the Fabrication of Organic OPV cells by inkjet printing of polymer:fullerene blends as photoactive layer [201,202,203,204]. Hoth, Brabec and co-workers reported inkjet formulations based on mixtures of high and lower boiling solvent (ortho-dichlorobenzene and 1,3,5-trimethylbenzene respectively) with reliable jetting behaviour, appropriate wetting, spreading and drying leading to smooth thin films with appropriate morphology and intermixing of polymer and fullerene phases. Photovoltaic devices prepared with these optimized formulations led to power conversion efficiencies in the range of 3% [202]. Studies carried out by the same group demonstrated that the control of the regioregularity of the polymer donor also plays a relevant role in the final morphology achieved and therefore in the performance of the device, allowing for further optimisation and reaching power conversion efficiencies of 3.5% [203]. Later on, Eom and co-workers further evaluated the influence of the addition of other high boiling point additives such as 1.8 octanedithiol, ortho-dichlorobenzene or chloronaphthalene to the photoactive layer ink based on chlorobenzene. As in the case of Hoth, morphology and solar cell performance were highly dependent on the presence of high boiling point additives reaching power conversion efficiencies of 3.71% [204]. Since morphology development of the photoactive layer is crucial for device performance, it is obvious that not only the composition, but also other inkjet printing processing parameters such as substrate temperature during printing or drying can be relevant for the performance of the printed photovoltaic devices [200,205,206].

Inkjet printing has also been used for the deposition of PEDOT:PSS as in the abovementioned case of OLEDs. The role of additives in device efficiency was explored by Eom and co-workers finding that the addition of glycerol and ethylene glycol butyl ether resulted in improved surface morphologies and conductivities leading to power conversion efficiencies up to 3.16% for solar cells with inkjet printed PEDOT:PSS layers [207]. As for the photoactive layer, substrate temperature or subsequent film annealing have also a large impact in the final morphology and therefore in the device performance [208]. The efficiency values attained in these devices are in the range of those obtained with other techniques such as spin or spray coating making inkjet very attractive for the preparation of PEDOT:PSS layers ubiquitous in OPV, as inkjet introduces a high degree of flexibility in the manufacturing process [209].

Apart from the hole transport layer, it is highly desirable for the R2R manufacturing of OPV solar cells that the rest of the electrodes can also be printed. ITO has usually been employed as transparent electrode in the preparation of solar cells. Although some attempts to prepare conductive ITO layers and patterns have been performed by inkjet printing of ITO nanoparticle formulations, they require, after deposition, a high temperature annealing (usually at temperatures higher than 450 °C) to reach the desired conductivity for OPV applications, making these approaches not compatible with flexible substrates [210]. In addition, as already mentioned in the OLED section, indium is a scarce element that increases the price of the photovoltaic device. It is brittle, compromising the performance and stability of devices and it presents relatively high sheet resistance, which limits the width of the active area of an individual solar cell [211]. As a result, the elimination of ITO from solar cell R2R fabrication is highly desirable and it is being intensively pursued. This issue is also relevant in the display industry and in general in flexible electronics, so the development of transparent alternative electrodes has pushed the research on different materials and strategies for this purpose. Inkjet printing of silver nanowires [212,213], carbon nanotubes [214] or graphene [215] has already been demonstrated. Besides novel materials, an interesting and cost-effective alternative consists in the application on a substrate of a highly conductive metallic mesh (e.g., Ag, Al or Cu), preferably by printing techniques, that partially allows for the transmission of light in combination with another transparent conductive material (e.g., PEDOT:PSS) [216]. Galagan and co-workers, at the Holst Centre in Eindhoven, implemented ITO-free photovoltaic devices by inkjet-printing current-collecting grids with a silver nanoparticle ink and a layer of high-conductivity PEDOT:PSS forming a composite anode. BHJ solar cells with an active area of 2 × 2 cm^2^ led to power conversion efficiencies of 1.54% [217]. Larger active areas have also been achieved with high power conversion efficiencies (8 cm^2^ active area cell with 2.34% efficiency) as shown by Huang and co-workers [218]. Aspects such as the sintering of the printed grids by thermal and laser based methodologies have also been evaluated and optimized [219]. The overall geometry and features of the grid and the width of the conductive tracks can be finely controlled through inkjet printing. Even embedding of the inkjet printed silver grids within the substrate has been demonstrated to lead to a smoother surface for the deposition of the PEDOT:PSS layer, resulting in an overall good photovoltaic performance [220]. All these aspects play an important role in the final device efficiency demonstrating the strength of inkjet digital manufacturing for the preparation of patterned electrodes. Also, the back electrodes have been successfully processed by using inkjet printing technology to implement OPV cells with conventional and inverted configurations reaching, in some of the cases, similar efficiencies similar to those obtained in solar cells with evaporated back electrodes [221,222,223,224,225].

When thinking about the massive production of solar cells in an industrial production environment, large amounts of ink are expected to be deposited per unit of time and therefore also large amounts of solvents need to be evaporated. A large effort is being made to eliminate halogenated solvents, typically used in research for OPV device preparation, from industrial inks trying to reduce environmental impact [226,227]. Chlorine-free solvent mixtures have been demonstrated to lead to device efficiencies closer to those reached when using chlorinated solvents in the application of the photoactive layer as shown by Lange and coworkers [226].

**Figure 23:**
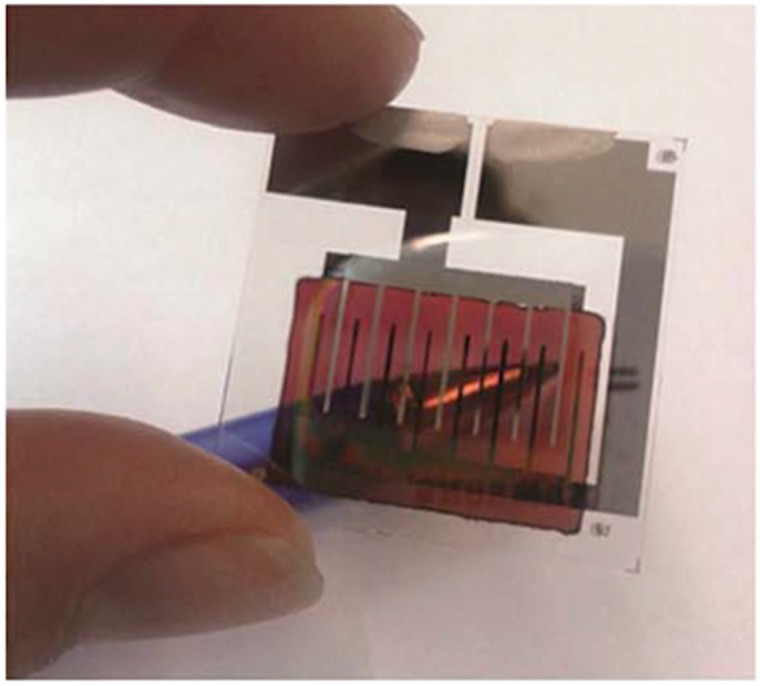
Semitransparent all-inkjet-printed solar cell with 1 cm^2^ active area. Reprinted from reference [229] with permission of The Royal Society of Chemistry.

As in the case of OLEDs, the fabrication of OPV multilayer devices by inkjet requires solvent compatibility with the underlying deposited layers in order not to deteriorate the device. Recently, Eggenhuisen and co-workers have shown the printing of four different layers using inkjet printing. Again, non-halogenated solvents have been used in the processing of the photoactive layer leading to devices with similar performance to those processed with chlorinated solvents. OPV modules with 92 cm^2^ active area and efficiency of 0.98% have been achieved [228]. Seeking the industrialisation of OPV solar cells, efforts have been made in the production of all-inkjet printed devices produced in air atmosphere. Recently Jung and co-workers reported power conversion efficiency of 2% for an all-inkjet-printed solar cell with a small active area of 0.5 cm^2^ [193]. Eggenhuisen and coworkers subsequently presented improved values with an all-inkjet-printed photovoltaic device with an active area larger than 1 cm^2^ and power conversion efficiency of 4.1%, all this using environmentally friendly solvents in ambient atmosphere (Figure 23) [229].

As described in this section, polymeric solar cells have been widely studied in combination with inkjet printing processing methodologies. Dye sensitized solar cells, that are also promising candidates for cheap production of PV modules, have been only recently explored in combination with R2R methodologies and in particular with inkjet printing [230,231,232]. Despite the impressive advances in the production of laboratory size polymeric photovoltaic devices showing good stability and efficiencies that demonstrate the potential of organic solar cell technology, the industrialisation and commercialisation of these devices is still to come. Inkjet printing has largely been demonstrated to be a solid candidate to replace or complement other techniques in R2R production of OPV and can contribute to the marketisation and future success of the OPV technology. Several companies (Merck, BASF, Plextronics, etc.) already commercialize organic semiconducting materials and research institutions, some of them mentioned in this review, and companies like BELECTRIC OPV GmbH are devoting large efforts to make large area inkjet printed organic solar cells closer to the market [233,234]. Reduction in cost of the materials and effective realisation of R2R production processes keeping a high efficiency and long lifetimes are required to make this technology penetrate the market.

## 7. Inkjet Printing of Self-Organizing Materials: Liquid Crystals and Colloidal Crystals

Top-down approaches make use of advanced tools, such as photolithographic, e-beam, ion-beam or inkjet printing setups, to generate patterns on top of or into a substrate. In contrast, bottom-up strategies rely on the ability of certain molecules or larger size entities to self-assemble, that is to autonomously organize, into defined structures or patterns through different kinds of interactions even if these are very weak [235]. The effective combination of both strategies, “bottom-up” and “top-down”, allows us to generate hierarchical structures crossing several length scales from the nanometre dimension of the molecule or self-assembling unit to the size of the device [236]. In this sense, the combination of self-organizing materials with inkjet printing represents an opportunity for the preparation of devices with different levels of organisation and complex functionality.

**Figure 24:**
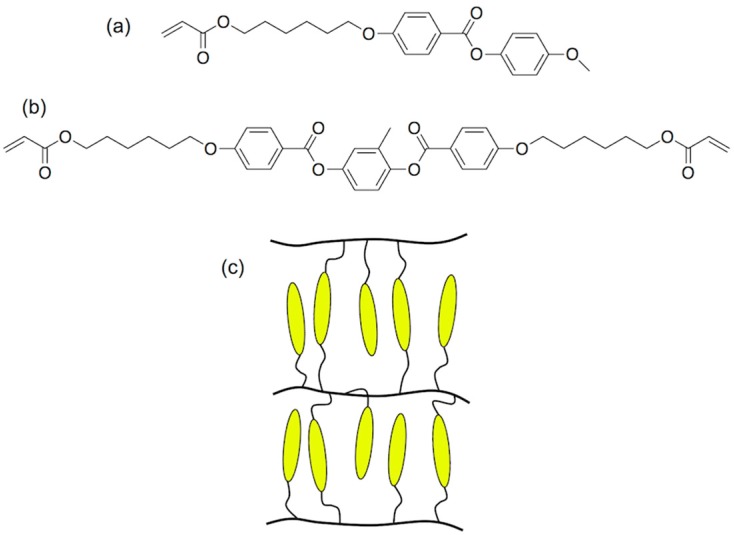
Reactive mesogens with (**a**) one or (**b**) two acrylate groups; (**c**) Diacrylate monomers establish crosslinks between polymeric chains.

Liquid crystals are a well-known example of self-organizing soft molecular materials showing orientational order that extends to macroscopic distances. Orientational order in these materials has its origin in the shape anisotropy of the constituent molecules that align around a common orientation named director. In principle, the direction of the director is arbitrary in space; however, in practice, this direction is dictated by external forces, even when these are very small. Liquid crystals can be aligned along small grooves in the substrate surface, by the action of external electrical or magnetic fields or by surface tension forces [237]. When liquid crystal molecules are provided with reactive groups such as acrylates, orientation of the liquid crystal can be frozen by in situ photopolymerisation [238]. Reactive mesogens, as the ones of Figure 24a,b, can be oriented in their low molecular weight state. By including a small percentage of a radical photoinitiator, irradiation with UV light starts polymerisation. The incorporation in the reactive mixture of monomers with two reactive diacrylate groups (Figure 24b) results in crosslinked systems (liquid crystalline elastomers or mesogenic networks) that can freeze the initial orientation achieved in the monomeric state, as shown in Figure 24c.

Inkjet printing of liquid crystalline materials containing photoisomerizable azobenzene moieties has been successfully employed in the processing of soft light-driven micro actuators. Irradiation with light in the absorption bands of the photoisomerizable azobenzene unit induces isomerisation and therefore local molecular disorder generating internal stresses in the material that lead to light controlled deformations [239]. Inkjet printing has also been used in the production of polymeric liquid crystalline optical films showing genuine polarisation effects that can be applied, as disclosed by Moia and Johnson, to the protection of documents or goods [240]. These liquid crystalline prints are only visible under special observation conditions using polarisers, acting as anti-counterfeiting features, as luminescent marks, that can also be obtained by inkjet printing, and are visible under certain light excitation conditions [241,242,243,244,245].

**Figure 25:**
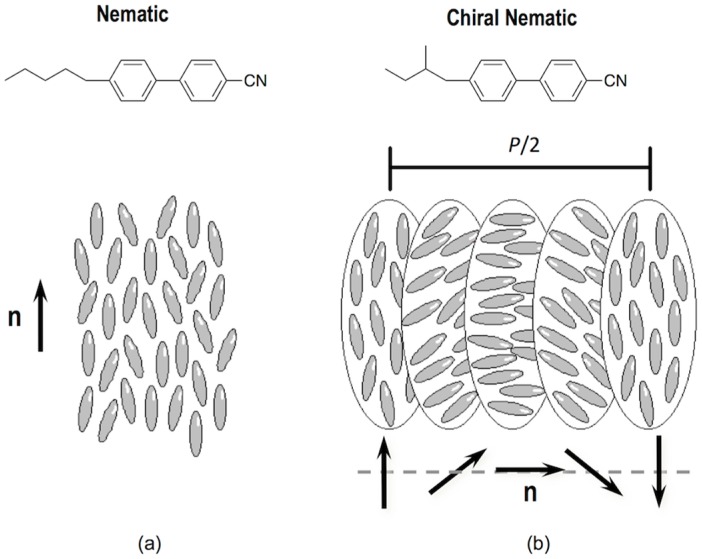
Chemical structure and mesophase arrangement of molecules in (**a**) a conventional nematic and in (**b**) a chiral nematic or cholesteric mesophase. The ability of inkjet printing to precisely deposit small amounts of material has been explored for the preparation of novel display concepts based on these cholesteric liquid crystals. Using a wall structure similar to that described in Section 4 and Section 5 of this review, a chiral material can be deposited into each individual well and be filled afterwards with a cholesteric gel. The resulting pixels reflect different colours depending of the deposited materials and the reflection properties can be electrically addressed forming a multicolour cholesteric liquid crystal display [247].

The inclusion of chiral molecules in a nematic liquid crystal induces the formation of a helical structure of the director having a pitch P that depends on the chiral dopant concentration and its helical twisting power (HTP) or ability to twist the nematic phase (Figure 25). Due to the optical anisotropy of the nematic phase, the introduced helical structure generates a periodic modulation of the refractive index leading to a photonic structure. These structures selectively reflect circularly polarised light of the same handedness of the helical structure and the reflected wavelength λ is given by the expression:

(7) $$\lambda =\frac{n}{HTP\cdot c}$$

where n is the mean refractive index, that is the average of the ordinary and extraordinary refractive index, and c is the concentration of chiral dopant in weight percent [246].

The photonic structure spontaneously formed in chiral nematic liquid crystals has also been used for the preparation of lasers. The inclusion of a luminescent chromophore in the photonic structure can lead to laser emission if the photonic band gap overlaps with the fluorescence maximum of the dye [248]. Manufacturing of LC laser arrays has been performed by Coles and Hutchings groups at the University of Cambridge. By inkjet printing of a dye containing cholesteric liquid crystal mixture onto a wet film of polyvinyl alcohol, round, uniform sessile droplets with the helical axis orthogonal to the substrate can be obtained. The inkjet deposited drops in this way showed laser emission under optical excitation at the maximum of absorption the dye, with very narrow linewidths below 1 nm and well defined laser thresholds [249].

This type of chiral structures has also been explored in the preparation of optical sensing devices for different types of stimuli [246,250,251]. A change in the molecular order, produced for example by temperature variation, can modify the cholesteric pitch and therefore the reflection wavelength becoming the system a temperature sensor. By including photoisomerizable molecules such as azobenzene chromophores, the cholesteric structure can act as a smart sensor that changes its colour with light. Light induced isomerisation of the azobenzene chromophores generates molecular disorder with a subsequent change in the helix pitch and reflected colour. Chemical sensors can also be prepared by the inclusion of selective receptor molecules into the chiral liquid crystalline material. Selective swelling of the helical structure can take place by selective molecular recognition, resulting in a change of the cholesteric reflection band.

**Figure 26:**
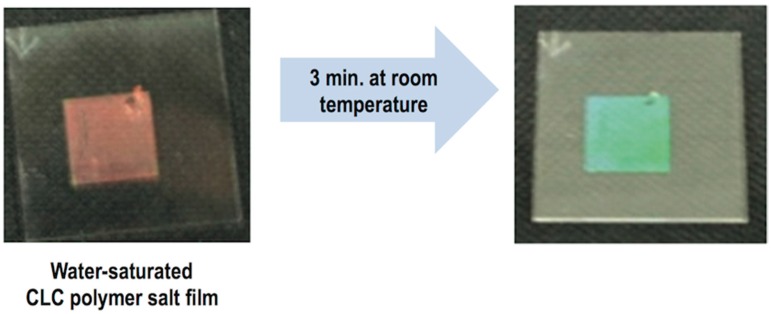
Inkjet printed water saturated cholesteric liquid crystal polymer salt film on polyimide coated glass in water (**left red film**) and after 3 min at room temperature (**right green film**). Adapted with permission from reference [253]. Copyright (2012) American Chemical Society.

Inkjet printing of humidity and temperature-sensitive films based on cholesteric liquid crystal layers has been demonstrated with potential application in sensing or anti-counterfeiting applications [252,253]. The work carried out by Schenning, Bastiaansen, Broer and co-workers has consistently demonstrated the preparation of battery-free sensors by inkjet printing of reactive mesogens and subsequent photopolymerisation. For example, films based on chiral nematic LC networks H-bonded through carboxylic acid groups, are converted to a hygroscopic polymer salt that shows a fast and reversibly change of their colour in response to humidity. Water saturated sensors based on this system can act as temperature/time integrators of interest for the control of the cold chain of food and pharmaceuticals (Figure 26) [253]. Inkjet printing has also been used in the preparation of other type of irreversible temperature/time integrators based this time in mechanical embossing of previously printed films, at temperatures above the glass transition temperature (T_g_), followed by quenching below this temperature [254]. Trimethylamine sensors have also been prepared by inkjet printing using hydrogen-bonded cholesteric liquid crystals [255].

All the research carried out in this topic demonstrates the feasibility of inkjet printing to produce this type of low-cost, battery-free cholesteric liquid crystal devices. The possibility to pattern the sensor materials and to generate different stimuli arrays in a single substrate/device makes inkjet printing an attractive tool for the massive production of sensors.

**Figure 27:**
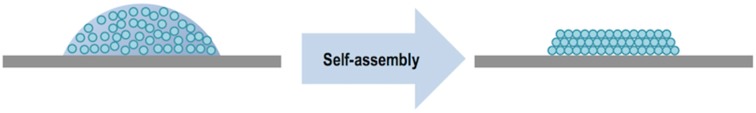
Monodisperse colloidal particles confined in a deposited drop self-assemble into well-ordered photonic structures during drying.

Besides liquid crystals, submicrometre-size, monodisperse colloidal particles can self-assemble into periodically well-organized structures that strongly interact with visible and IR light [256,257]. Compared to photolithographies, self-assembly offers a low-cost route for the manufacturing of these photonic structures over large areas, having the additional advantage of the easy inclusion of functionalities through their constituent colloidal particles. Patterning of these photonic structures has attracted extensive interest due to their potential application in the preparation of full colour displays, light waveguides, microfluidic devices or sensing arrays. Combination of bottom-up self-organisation of colloidal particles with top-down approaches has been explored to attain these patterned well-organised structures with hierarchical order. In particular, inkjet printing of colloidal dispersions has demonstrated to be an interesting approach for the patterned growth of colloidal photonic crystals. Patterns deposited by inkjet become the template for colloidal self-assembly, which is a highly precise, high-throughput scalable approach for the preparation of patterned photonic structures (Figure 27).

Fan and co-workers demonstrated the fabrication of hierarchically organized structures by an evaporation-induced self-assembly in combination with ink-jet printing [258]. They prepared an ink composed of oligomeric silica sols in ethanol/water with a surfactant concentration well below the critical micelle concentration (c.m.c.). After drop deposition by inkjet printing onto a surface, evaporation of ethanol makes the surfactant exceed the c.m.c., especially at the liquid-vapour interfaces, thus triggering the formation of micelles and their self-organization into long-range periodic structures. The possibility of generating microstructures with hierarchical organisation at the nanometre scale was demonstrated to be of interest in the fabrication of pH sensors for fluidic systems and with potential application in photonic devices and displays.

Besides the printing of silica sols, Moon and co-workers demonstrated the formation of photonic structures by evaporation-induced self-assembly of monodisperse microspheres of silica or polystyrene deposited as droplets by inkjet printing [259,260,261,262]. Morphology and size of the colloidal crystals can be controlled by adjusting the wettability of the substrate and the ink composition [259,260,261,262,263,264,265]. For example, evaporation of microsphere containing droplets showing a high contact angle, usually leads to dome like colloidal crystals of small radius, compared with the initial deposited drop size, since the contact line freely recedes without changing the angle. On the other hand, low contact angles usually lead to colloidal crystals with ring-like morphology. In this last case the liquid layer is very thin at the contact line and evaporation produces pinning of particles at the three-phase contact line. Further evaporation with this pinned contact line prevents the receding of the drop and drags the suspension toward the outer region, leading to concentration of the colloidal particles in the ring-like geometry, that is the coffee stain effect mentioned in Section 2 [259]. As in the case of polymer solutions, this effect can be suppressed or controlled by using a rationally designed mixture of solvents. Convective flow of particles towards the contact line can be compensated by the Marangoni flow induced by the surface tension gradient obtained when a small amount of higher boiling point/lower surface tension solvent is added to a lower boiling point/higher surface tension majority ink solvent. This can lead to homogenous round-shaped photonic crystals for isolated deposited droplets or line-shaped photonic structures when a continuous line of colloidal ink is deposited [261].

Another further development was carried out by Song´s group when they printed large area patterned photonic crystals by ink-jet printing technology [266]. They used a polymer latex suspension of core-shell microspheres having a polystyrene core and a hydrophilic and soft poly(methyl methacrylate)/polyacrylic acid shell that favours self-assembly. By printing colloidal inks with microspheres of different diameter, namely 280, 220 and 180 nm, patterned photonic crystals showing respectively light reflections centred at 646, 541 and 465 nm, that is red, green and blue light were demonstrated. As in previous works from Moon, an adequate wettability of the substrate and the introduction of a co-solvent as ethylene glycol revealed to be crucial to obtain homogeneous photonic deposits of high quality. This facile fabrication method of patterned photonic crystals by ink-jet printing is promising for the preparation of photonic devices and optical circuits. Anti-counterfeiting holograms for documents and goods protection have also been recently demonstrated by Nam and co-workers by using this technique [267].

**Figure 28:**
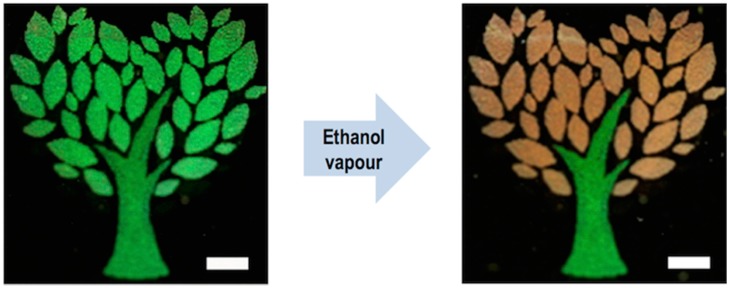
Inkjet printed colloidal photonic crystal pattern. The printed leaves are composed of self-assembled mesoporous silica microparticles with large adsorption capability while the tree trunk is made of solid silica microparticles. When exposed to N_2_ (**left**) both photonic crystals present the same reflected colour. Exposure to ethanol vapour (**right**) reveals a change of colour in the mesoporous colloidal photonic crystal. Scale bar: 0.5 cm. Adapted with permission from Reference [269]. Copyright 2014 American Chemical Society.

As in the case of liquid crystalline cholesteric photonic structures, the colloidal photonic crystals can be made responsive to external stimuli by incorporation of appropriate functionalities in the structure. Colloidal photonic crystals prepared using inkjet printing have been employed in the implementation of humidity, vapour or protein sensors [268,269,270]. As an example, the group of Song has demonstrated the fabrication by inkjet printing of humidity sensors based on photonic crystals. The inclusion into the photonic crystal of poly(N-isopropyl acrylamide), presenting a reversible phase transition between a collapsed dehydrated state to a swollen hydrated form, is responsible of the fast and visible colour change in the order of seconds in response to the presence of water vapour [268]. Vapour responsive multicolour patterns were also reported by Bai and co-workers. They inkjet printed mesoporous silica microparticles with large active surface area and also large adsorption capability that can self-assemble forming colloidal photonic crystals. Vapour adsorption leads to changes in the average refractive index and therefore different reflection of the photonic structures as shown in Figure 28 [269].

Finally, inkjet printing can also be carried out on top of photonic crystals structures previously prepared by other methodologies locally varying the optical properties in these regions. This strategy has recently been used in the preparation of sensors for metal ions recognition [271]. Besides sensing, when droplets of other materials are infused into the photonic crystal, local swelling takes place in these regions, changing the reflected colours, an effect that has been used in the preparation of high resolution multicolour images [272].

Summarizing this section, the combination of self-assembly and inkjet printing has demonstrated to be an interesting approach for the preparation of functional surfaces and devices. Liquid crystal and colloidal microparticle based inks can be deposited by inkjet printing leading to hierarchical structures showing special optical behaviour. Control of the chemical functionality and the optical properties of the deposited materials as well as of the deposition process allows us to implement complex optical components as well as photonic devices such as lasers, sensors, anticounterfeiting elements or displays.

## 8. Summary and Conclusions

Beyond its use in graphics and marking, inkjet printing has become a very attractive tool for the digital manufacturing of functional surfaces and devices due to its ability to precisely position, with no-contact, a wide range of materials on a manifold of substrates. Besides this flexibility in pattern formation, inkjet printing is an additive method that makes a very efficient use of materials, being therefore an environmentally friendly technique. Although inkjet printing is a serial patterning process, it can be scaled up by using multi-nozzle multi-heads with different inks allowing for the manufacturing of complex multi-material patterns with high throughput. The large interest in this technology has fed research in the fundamental physical processes that encompasses the generation of droplets, the interaction of the droplet with the substrate and the final transformation of the ink into a solid material. Understanding the relation between printhead architecture and dynamics, the acoustics and jet formation has allowed us to gain control on the drop generation process. The printability of different fluids has been largely explored identifying favourable regimes for stable drop formation at certain combinations of fluid properties (mainly viscosity, surface tension and density). However, as more demanding applications require the deposition of inks carrying polymers and/or microparticles with complex rheological behaviour, more research needs to be done to understand the drop formation mechanism in these fluids.

This review has presented an overview of the use of inkjet printing in the preparation of different optical elements and photonic devices. Inkjet printing has extensively been demonstrated to be a valuable tool for the manufacturing of microlenses with accurate control of the final optical parameters through appropriate selection of used materials and wetting characteristics of the substrate. Much less work has been carried out in the field of integrated optics despite interesting advances in the fabrication, using inkjet technology, of planar waveguides, inter-board optical connections between polymeric waveguides as well as integrated microring lasers have been done. Compared to other competitor technologies, such as photolithographies, inkjet printing has demonstrated to require less processing steps potentially being an interesting approach for the effective fabrication and integration of optical micro-devices.

In the field of large area photonic devices, a great effort has been done over the last two decades in the development of solution-processable materials for display and organic photovoltaic devices. The production using inkjet printing technology of OLED devices with excellent performance in terms of efficiency and durability, as well as colour gamut, as in the case of devices incorporating QDs, has been demonstrated in the laboratory. Efficient and stable BHJ solar cells have also been successfully produced at small scales by using this printing technology. Large efforts are being made to effectively integrate inkjet printing of these materials with R2R fabrication to reach high-efficiency and low-cost devices and make them closer to the market. Thinking in large-scale production, environmental issues are especially relevant, and attempts to eliminate chlorinated solvents of the production process are being done. Already, several small and large companies, covering the whole value chain, are already involved in finding a way towards the industrial production of large area photonic devices. Chemical companies commercialize organic semiconducting materials and industrial inkjet printing equipment with reasonably high throughput and yield is being developed specifically for these applications. Overall, inkjet printing technology appears to be a key enabling technology for the industrialisation and commercialisation of large area photonic devices in the near future.

Finally, the combined use of self-organizing materials and inkjet printing technology has been reviewed. In particular the use of liquid crystal and colloidal microparticle based inks has demonstrated to be an interesting approach for the preparation of hierarchical structures with striking optical behaviour. Introduction of specific functionalities in the inks has allowed us to implement photonic devices such as lasers or battery-free sensors for light, temperature or chemical vapour detection.

As an overall conclusion, inkjet printing has been demonstrated to be a valuable tool for the preparation of different optical components and photonic devices. As applications become closer to the market, requirements for inkjet printing are more demanding in terms of the used materials and also in the technology itself. Materials and processes resulting in cheaper and better performance devices are needed. More complex and extreme fluids, such as polymer solutions or colloidal dispersions, are nowadays being used requiring each time better performance printheads as well as a better understanding of fluid properties covering aspects that go from drop formation to its interaction with the substrate and fixation. All this makes the field of inkjet printing research an extremely active and challenging area with fundamental research and industrial development advancing together towards applications.
